# Repositioning Lopinavir, an HIV Protease Inhibitor, as a Promising Antifungal Drug: Lessons Learned from *Candida albicans*—In Silico, In Vitro and In Vivo Approaches

**DOI:** 10.3390/jof7060424

**Published:** 2021-05-28

**Authors:** André L. S. Santos, Lys A. Braga-Silva, Diego S. Gonçalves, Lívia S. Ramos, Simone S. C. Oliveira, Lucieri O. P. Souza, Vanessa S. Oliveira, Roberto D. Lins, Marcia R. Pinto, Julian E. Muñoz, Carlos P. Taborda, Marta H. Branquinha

**Affiliations:** 1 Laboratório de Estudos Avançados de Microrganismos Emergentes e Resistentes (LEAMER), Departamento de Microbiologia Geral, Instituto de Microbiologia Paulo de Góes (IMPG), Universidade Federal do Rio de Janeiro (UFRJ), Rio de Janeiro 21941-901, Brazil; lysmonica@yahoo.com.br (L.A.B.-S.); fusariumsp@gmail.com (D.S.G.); liviaramos2@yahoo.com.br (L.S.R.); simonesantiagorj@yahoo.com.br (S.S.C.O.); lucieri@micro.ufrj.br (L.O.P.S.); 2 Programa de Pós-Graduação em Bioquímica (PPGBq), Instituto de Química (IQ), Universidade Federal do Rio de Janeiro (UFRJ), Rio de Janeiro 21941-909, Brazil; 3 Instituto Aggeu Magalhães, Fundação Oswaldo Cruz, Recife 50740-465, Brazil; vanessa.silvao@ufpe.br (V.S.O.); roberto.lins@cpqam.fiocruz.br (R.D.L.); 4 Departamento de Microbiologia e Parasitologia, Instituto Biomédico, Universidade Federal Fluminense (UFF), Niterói 24210-130, Brazil; mpinto@id.uff.br; 5 MICROS Group, Medicine Traslacional Institute, School of Medicine and Health Sciences, Universidad del Rosario, Bogotá 111221, Colombia; juliane.munoz@urosario.edu.co; 6 Departamento de Microbiologia, Instituto de Ciências Biomédicas, Universidade de São Paulo (USP), São Paulo 05508-060, Brazil; taborda@usp.br; 7 Laboratório de Micologia Médica—LIM53/IMTSP, Universidade de São Paulo (USP), São Paulo 05508-000, Brazil

**Keywords:** *Candida albicans*, drug repurposing, HIV protease inhibitors, secreted aspartic proteases, lopinavir, virulence, adhesion, biofilm, morphogenesis, animal infection

## Abstract

The repurposing strategy was applied herein to evaluate the effects of lopinavir, an aspartic protease inhibitor currently used in the treatment of HIV-infected individuals, on the globally widespread opportunistic human fungal pathogen *Candida albicans* by using in silico, in vitro and in vivo approaches in order to decipher its targets on fungal cells and its antifungal mechanisms of action. Secreted aspartic proteases (Saps) are the obviously main target of lopinavir. To confirm this hypothesis, molecular docking assays revealed that lopinavir bound to the Sap2 catalytic site of *C. albicans* as well as inhibited the Sap hydrolytic activity in a typically dose-dependent manner. The inhibition of Saps culminated in the inability of *C. albicans* yeasts to assimilate the unique nitrogen source (albumin) available in the culture medium, culminating with fungal growth inhibition (IC_50_ = 39.8 µM). The antifungal action of lopinavir was corroborated by distinct microscopy analyses, which evidenced drastic and irreversible changes in the morphology that justified the fungal death. Furthermore, our results revealed that lopinavir was able to (i) arrest the yeasts-into-hyphae transformation, (ii) disturb the synthesis of neutral lipids, including ergosterol, (iii) modulate the surface-located molecules, such as Saps and mannose-, sialic acid- and *N*-acetylglucosamine-containing glycoconjugates, (iv) diminish the secretion of hydrolytic enzymes, such as Saps and esterase, (v) negatively influence the biofilm formation on polystyrene surface, (vi) block the in vitro adhesion to epithelial cells, (vii) contain the in vivo infection in both immunocompetent and immunosuppressed mice and (viii) reduce the Sap production by yeasts recovered from kidneys of infected animals. Conclusively, the exposed results highlight that lopinavir may be used as a promising repurposing drug against *C. albicans* infection as well as may be used as a lead compound for the development of novel antifungal drugs.

## 1. Introduction

Mucosal candidiasis, including oropharyngeal, esophageal and vaginal candidiasis, is common among human immunodeficiency virus (HIV)-infected individuals, and the risk is considered to be higher in patients with CD4^+^ T lymphocytes counts of <200 cells/mm^3^ and high plasma HIV RNA loads [1,2,3,4,5,6,7,8]. Due to its relevance, oropharyngeal candidiasis is a sentinel indicator of immunodeficiency and HIV disease progression [9]. Deep-seated candidal infections are an important cause of morbidity and mortality in immunocompromised individuals and severely ill patients [7]. *Candida albicans* represents the most common causative agent of oral, vaginal and disseminated candidiasis [4,7]. Although the fungus is a harmless commensal in healthy individuals, *C. albicans* can rapidly proliferate, invade tissues and cause symptomatic mucosal lesions in immunocompromised persons. *C. albicans* is the main widespread human fungal pathogen, which is commonly recovered from hospital settings around the globe. *C. albicans* possesses a genomic plasticity that directly reflects in a metabolic diversity and in the production of a plethora of virulence attributes, which helps it to colonize and adapt to several distinct physicochemical environments as well as to escape from host immune responses [7,8]. These virulence attributes include the (i) surface adhesin molecules (e.g., mannoproteins and glycoconjugates); (ii) reversible transition between unicellular yeast cells into filamentous forms (morphogenesis process); (iii) ability to form robust biofilm on both inert and living surfaces and (iv) secretion of extracellular hydrolytic enzymes (e.g., proteases and lipases) [7,8].

Remarkable findings from clinical trials with HIV-infected populations have shown a decrease in oral candidal infection as well as other classical oral manifestations of HIV infection [9,10,11,12]. Oral candidiasis decrease was first observed when antiretrovirals belonging to the protease inhibitor (PI) class were included amongst the drugs used to treat HIV infection in the well-known highly active antiretroviral therapy (HAART), and this mucocutaneous infection is nowadays only rarely observed in treated patients [9,10,11,12]. Considered a very effective therapy, HAART decreases the HIV viral load and leads to the increase in CD4^+^ T lymphocyte counts and the partial recovery of T cell responses against recall antigens, as well as stimulates the survival and activation of neutrophil, monocyte, dendritic and endothelial cells [13,14,15,16], causing an improvement in the immunity and a decrease in the incidence of opportunistic infections [17,18]. For instance, the treatment with saquinavir leads to the resolution of mucosal candidiasis in a patient refractory to the treatment with fluconazole [19]. However, controversy exists concerning whether the decrease in frequency of candidiasis in HIV patients is due to an improvement in the patient’s immune status, or to an effect of the HIV PIs on the colonization capacity of *C. albicans*, or to an antifungal effect exerted by HIV PIs. This hypothesis was based on clinical observations in which HIV-positive subjects receiving HAART manifest a marked decrease in the incidence of oral candidiasis, sometimes soon after initiation of therapy and well before the number of CD4^+^ T lymphocytes recover [20]. Subsequently, several lines of evidence have proposed that the HIV PIs have direct inhibitory effects on *C. albicans* aspartic proteases (designated as secreted aspartic proteases—Saps, which are key virulence factors), culminating in the reduction of fungal growth [21]. The *C. albicans* Saps are thought to play a multimodal role in the infectious process; so, the development of Sap inhibitors for the treatment of candidiasis thus appears to be a valid strategy [21].

The antifungal activity of the first-generation HIV PIs (e.g., ritonavir, saquinavir, indinavir and ritonavir) was demonstrated in in vitro and in in vivo studies, particularly against yeasts (e.g., *Candida* spp. and *Cryptococcus neoformans*) and some filamentous fungi (e.g., *Fonsecaea pedrosoi* and *Pneumocystis jirovecii*) [21,22]. However, contradictory results regarding the antifungal action of HIV PIs have been published in the literature. A simple explanation by those ambiguous results is based on the different methodologies that were employed; for example, to detect the antifungal activity in which distinct culture medium, time of fungal-drug exposure, drug concentration, number of fungi tested and method to reveal the experiment. In addition, the second generation of HIV PIs presents better pharmacological (pharmacokinetics/pharmacodynamics) parameters. These novel HIV PI treatment regimens attained preferred status because they have clinical trial data support for their efficacy, durability of effect, tolerability and ease of use [23]. For instance, Otto et al. [24] described a case of cryptococcal meningitis associated with an immune reconstitution inflammatory syndrome (IRIS) in a ten-year-old HIV-infected child prior to the switch from the first-line to second-line HAART (abacavir-lamivudine efavirenz to zidovudine-lamivudine-lopinavir/ritonavir) due to the virological failure. So, the repurposing of HIV PIs as antifungal agents is a valid and contemporary strategy.

In the present work, lopinavir, an HIV PI commonly used in the clinical arena to treat HIV-infected patients, was evaluated against the ubiquitous opportunistic human fungal pathogen *C. albicans* by multimodal strategies using in silico, in vitro and in vivo experimental approaches in order to decipher its targets on fungal cells and its antifungal mechanisms of action.

## 2. Materials and Methods

### 2.1. Chemicals

Lopinavir was obtained from the National Institutes of Health (NIH) and dissolved in dimethylsulfoxide (DMSO) to obtain a final concentration of 20 mM. Bovine serum albumin (BSA), pepstatin A, ergosterol, lanosterol, fluorescein isothiocyanate (FITC)-labeled secondary antibody, FITC-Concanavalin A (Con A), FITC-*Limax flavus* agglutinin (LFA), FITC-wheat germ agglutinin (WGA), Calcofluor white, crystal violet, menadione, 2,3-bis (2-methoxy-4-nitro-5-sulfophenyl)-5-[(phenylamino) carbonyl]-2H-tetrazolium hydroxide (XTT), formalin, Dulbecco’s Modified Eagle’s Medium (DMEM), Giemsa and cyclophosphamide were purchased from Sigma-Aldrich (St. Louis, MO, USA). Yeast carbon base (YCB) and brain and heart infusion (BHI) media were obtained from HiMedia Laboratories Ltd. (Mumbai, India). Fetal bovine serum (FBS) was purchased from Cultilab (São Paulo, Brazil). Ketamine and xylazine were obtained from União Química Farmacêutica (São Paulo, Brazil). Reagents used in microscopy, electrophoresis and buffer components were purchased from Amersham (Little Chalfont, United Kingdom). All other reagents were of analytical grade.

### 2.2. Fungal Strain and Growth Conditions

*Candida albicans* strain 11 was previously isolated by our research group from the blood of an HIV-positive, 46-year-old male. This strain presents a trailing phenomenon in terms of an in vitro susceptibility test with azoles, and expresses a high amount of both cell-associated and extracellular aspartic-type proteases, particularly Sap2 (Figure S1) [25]. In all experiments, fungal cells were cultivated on 1.2% YCB supplemented with 0.1% BSA, pH 5.0, and grown at 37 °C for 48 h under slight agitation (100 rpm), which is a well-known Sap-inducing condition [25,26,27]. Cell growth was estimated by counting the yeasts in a Neubauer chamber.

### 2.3. Effects of Lopinavir on C. albicans Sap2: In Silico Approach

Molecular docking assays of the standard ligand A70450, a synthetic hexapeptide analogue to pepstatin A (Figure S2), and lopinavir to the active site of the *C. albicans* Sap2 protein were carried out using the Autodock Vina 1.1.2 program [28]. The atomic coordinates for Sap2, complexed with its ligand, were taken from the Protein Data Bank (PDB ID 1EAG) [29]. The ligand coordinates were removed and the pKa of each titratable residue estimated by the Propka 3.0 program [30]. Except for D_32_ that presented an estimated pKa of 9.88, all the remaining residues showed no significant shifts from their model reference values. Due to the uncertainty regarding the protonation states of the nitrogen atoms in lopinavir, two protonation states for lopinavir were considered (Figure S2). Atomic charges for protein and ligands were assigned using the Gasteiger method in the ADT 1.5.4 program [31]. A grid box, encompassing the center of the mass of residues D_32_, D_218_ and G_85_ of dimensions 22 × 22 × 22 Å, was used for the molecular docking. These residues were chosen due to their identified hydrogen-bond interactions with A70450. A total of 9 binding modes were generated in each case, with an estimated 3 kcal/mol maximum difference between the best and worst binding modes. The associated error of the method is typically 1–2 kcal/mol. Therefore, the energetic criterion, the raking (most prevalent conformation) and the interactions of the ligand with the catalytic dyad and G_85_ were analyzed.

### 2.4. Effects of Lopinavir on Saps: In Vitro Assay

In order to obtain the conditioned supernatant of *C. albicans* cells, which is rich in Saps (Figure S1), the 96 h-old culture was harvested by centrifugation at 4000× *g* for 10 min at 4 °C, and the supernatant was filtered in a 0.22-µm membrane (Millipore, São Paulo, Brazil) and used to measure the extracellular protease activity. Proteolytic activity was measured spectrophotometrically using the substrate BSA, according to the method described by Buroker-Kilgore and Wang [32]. Initially, the supernatant fluid was incubated for 30 min at 37 °C in the presence or in the absence of 10 µM pepstatin A (a classical aspartic PI) or lopinavir at different concentrations ranging from 6.25 to 200 µM. For assaying the remaining proteolytic activity, BSA (0.5 mg/mL), 20 mM sodium citrate (pH 4.0) and 40 µL of supernatant fluid were added to a microcentrifuge tube (350 µL) and incubated for 2 h at 37 °C. After this incubation, three aliquots (100 µL each) of the reaction mixture were transferred to wells on a microtiter plate containing 50 µL of water and 100 µL of a Coomassie solution (0.025% Coomassie brilliant blue G-250, 11.75% ethanol and 21.25% phosphoric acid). After 10 min to allow dye binding, the plate was read on a Thermomax Molecular Device microplate reader at an absorbance of 595 nm.

### 2.5. Effects of Lopinavir on Growth

In this set of experiments, two distinct approaches were performed to study the effects of lopinavir on *C. albicans* when grown in YCB-BSA medium: (i) different densities of yeasts (10^2^ to 10^6^ cells) were incubated with lopinavir at 50 µM, and (ii) 10^3^ yeasts were treated with different lopinavir concentrations (3.125 to 100 µM). All the systems were incubated for 20 h at 37 °C with slight agitation (100 rpm). Subsequently, fungal cells were washed three times in phosphate-buffered saline (PBS, pH 7.0) and re-inoculated in BHI medium without drugs, to measure the colony-forming units (CFUs) [33]. The solvent (DMSO) has also been tested for its possible ability to inhibit cellular growth, presenting negative results. A control was also made replacing lopinavir with PBS.

### 2.6. Lopinavir Treatment: Looking for Possible Molecular Targets in C. albicans Cells Other Than Saps and Role in Crucial Physiopathology Events

For all the subsequent experiments (except when discriminated), 10^6^ yeast cells were treated (or not) with lopinavir (at both 100 and 200 µM) for 20 h in order to detect the influence of this HIV PI on the (i) morphological/ultrastructural architecture; (ii) morphogenesis; (iii) expression of surface molecules; (iv) production of extracellular enzymes; (v) interaction with abiotic (polystyrene) and biotic (epithelial cells) surfaces and (vi) infection using murine models.

### 2.7. Effects of Lopinavir on Morphometrical Parameters and Morphology

Untreated and lopinavir-treated fungal cells were fixed with 4% paraformaldehyde at 4 °C for 30 min and then analyzed in a flow cytometer (FACSCalibur; BD Bioscience, La Jolla, CA, USA) equipped with a 15-mW argon laser emitting at 488 nm. Each experimental population was mapped (10,000 events) using a two-parameter histogram of forward-angle light scatter (FSC) versus side scatter (SSC) in order to evaluate size and granularity, respectively. In parallel, fungal cells were incubated with 100 µL Calcofluor white solution (1 mg/mL) and incubated for 5 min at room temperature. Then, cells were washed three times with PBS and resuspended in 100 µL of the same buffer. Five microliters of the stained cell suspension were spotted onto a glass slide and visualized under a bright field and UV/DAPI using an Observer Z1 (Zeiss, Jena, Germany) fluorescence microscope. Images were acquired with a Color View AxioCam MRm digital camera.

### 2.8. Effects of Lopinavir on Ultrastructural Architecture

In this set of experiments, both transmission (TEM) and scanning (SEM) electron microscopies were performed in order to detect fungal ultrastructural changes. For TEM assay, fungal cells were fixed with a solution containing 2.5% glutaraldehyde, 4% paraformaldehyde and 10 mM CaCl_2_ in 0.1 M cacodylate buffer (pH 7.2) for 1 h at 25 °C. Then, fungal cells were washed with the same buffer and post-fixed for 1 h in a solution containing 1% OsO_4_ and 0.8% potassium ferricyanide in cacodylate buffer. Finally, the cells were rinsed, dehydrated in a graded series of acetone and embedded in Spurr resin. Ultrathin sections were obtained, stained with uranyl acetate and lead citrate and examined using a JEOL 1200 EX transmission electron microscope [34]. For SEM analyses, fungal cells were washed in cacodylate buffer (0.1 M) and fixed for 2 h at room temperature in the same buffer containing 2.5% glutaraldehyde. Then, fungi were post-fixed in 1% OsO_4_ for 1 h at room temperature and adhered to poly-l-lysine-coated glass coverslips (0.1%). Fungi were then washed three times with PBS and then dehydrated in increasing ethanol series (30, 50, 70, 80, 95 and 100%). After this step, the preparations were dried by the critical point method, mounted on aluminum supports and coated with gold-palladium. Electron micrographs were obtained from the Quanta 250 scanning electron microscope (FEI Company).

### 2.9. Effects of Lopinavir on Morphogenesis

The transformation of yeasts into germ tubes (morphogenesis process) was performed by incubating 10^6^ yeasts in 1 mL of FBS [35,36] supplemented or not with different concentrations of lopinavir (varying from 12.5 to 200 µM). All the systems were incubated for 4 h at 37 °C without agitation. At the end of the incubation period, samples were used for microscopic assessment of germ tube production in a Zeiss microscope (Zeiss). The percentage of germination was estimated by counting 100 fungal cells in a Neubauer chamber.

### 2.10. Effects of Lopinavir on Neutral Lipids

#### 2.10.1. Sterol Content

Fungal cells were subjected to incubation with a chloroform:methanol mixture (2:1 *v*/*v*) in order to extract the lipids [37]. Precipitated material was removed by centrifugation, and the solution was then reduced to dryness under nitrogen. Following Folch partition [38], the sterol content of the lipid extract was analyzed by high-performance thin-layer chromatography (HPTLC) using a solvent mixture of hexane-ether-acetic acid (80:40:2 *v*/*v*/*v*) and revealed a solution comprising 50 mg of FeCl_3_, 90 mL of water, 5 mL of acetic acid and 5 mL of H_2_SO_4_. The plate was heated to 100 °C for 5 min, and the bands corresponding to sterols were then visualized and compared to sterol standards, ergosterol and lanosterol [39]. In order to quantify the ergosterol in the chromatogram more accurately, the plate was initially digitized, and the bands corresponding to the ergosterol migration were manually selected using the free selection tool provided by the Image J software. Band areas were then determined by repeating this process 3 times, to diminish the probability of errors in these estimations. Values of band area were further integrated with means of grey level in selected bands, generating densitometric values (which were expressed as arbitrary units—AU) that were used in the comparison between corresponding bands in the same chromatogram [40].

#### 2.10.2. Nile Red Staining

Fungi were incubated with Nile red (5 μg/mL) in the dark for 30 min for the detection of lipid bodies, a place of accumulation of intracellular neutral lipids, by both fluorescence microscopy and flow cytometer assays. For fluorescence microscopy, the fungal cells were deposited in blade/coverslip systems and observed with a fluorescence microscope Zeiss Axioplan-D image D2. In the flow cytometry assay, the mapped population was analyzed for log red fluorescence using a single-parameter histogram, and the results were expressed as the percentage of fluorescent cells (%FC) and mean of fluorescence intensity (MFI).

### 2.11. Effects of Lopinavir on Cell Wall-Located Surface Molecules

In this set of fluorocytometric analyses, fungal cells were initially fixed in 4% paraformaldehyde for 1 h, rinsed in PBS and then incubated for 1 h at room temperature in the presence of the following fluorescent agglutinins: FITC-Con A (5 µg/mL), FITC-WGA (10 µg/mL) and FITC-LFA (25 µg/mL), which recognize α-D-mannosyl/α-D-glucosyl residues, *N*-acetylglucosamine oligomers and *N*-acetylneuraminic acid, respectively [41]. In addition, the anti-Sap1-3 polyclonal antibody raised against *C. albicans* (kindly provided by Dr. Nina Agabian, University of California, San Francisco, CA, USA), at 1:1000 dilution, was also tested to detect surface-located aspartic proteases (Saps). Fungi were washed in PBS and subsequently treated with the primary antibody followed by a 1 h incubation period with the FITC-secondary antibody. In parallel, fungal cells that had not been incubated with agglutinins or antibodies were also prepared in order to run as autofluorescence control systems. Finally, fungi were washed in PBS and analyzed by flow cytometry. Control cells were analyzed first in order to determine their autofluorescence and relative size. The mapped population (10,000 events) was analyzed for log green fluorescence by using a single-parameter histogram. The results were expressed by means of two parameters: %FC and MFI.

### 2.12. Effects of Lopinavir on Secreted Hydrolytic Enzymes

Two major groups of hydrolytic enzymes produced by *C. albicans* cells were evaluated: proteases and lipases. Aspartic protease production was assayed using the albumin agar plate (1.17% YCB medium supplemented with 0.2% BSA, pH 4.0) as previously described by Ruchel et al. [42]. The protease activity results in a clear zone around the colony, which corresponds to the hydrolysis of the BSA present in the medium. In parallel, the production of two distinct lipases, phospholipase and esterase, was also investigated. The determination of phospholipase activity was performed using an egg yolk agar plate (1 M NaCl, 5 mM CaCl_2_ and 8% sterile egg yolk emulsion, pH 7.0) according to Price et al. [43]. The esterase production was assayed using the Tween agar plate (1 g of peptone, 0.5 g of NaCl, 0.01 g of CaCl_2_, pH 7.0, 1.5 g of agar and 100 mL of distilled water, which was autoclaved, then cooled to about 50 °C, and 0.5 mL of autoclaved Tween were added) according to Aktas et al. [44]. In these methods, the hydrolysis of lipid substrates present in egg yolk or Tween results in the formation of a calcium complex with fatty acids released by the action of the secreted enzymes, resulting in a precipitation zone around the colony. The colony diameter (a) and the diameter of colony plus precipitation zone (b) were measured by a digital paquimeter. The enzymatic activities were expressed as *Pz* values (a/b) as described by Price et al. [43]. According to this definition, low *Pz* values mean high enzymatic production, and, inversely, high *Pz* values indicate low enzymatic production.

### 2.13. Effects of Lopinavir on Biofilm

The effects of lopinavir were tested on different biofilm perspectives: (i) pre-treatment, when fungal cells (10^6^ yeasts) were pre-treated or not with lopinavir at 100 or 200 μM for 20 h and then incubated for 48 h over a polystyrene substrate; (ii) joined treatment, when fungal cells were plated at the same time to adhere to the polystyrene substrate in the absence or in the presence of lopinavir (100 and 200 μM), and then the systems were incubated for 48 h; and (iii) post-treatment, when fungal cells were first adhered to polystyrene for 48 h, and then the mature biofilm was treated with lopinavir (100 and 200 μM) for an additional 20 h. After all these protocols, the spent medium was aspirated, and non-adherent cells were removed by thoroughly washing the biofilms with PBS. Wells with media and no cells were included as blank wells. Finally, two biofilm parameters were analyzed: biomass and viability (metabolic activity). The biomass of the resultant biofilms was then assessed using the crystal violet assay [45]. Briefly, 100 µL of 99% methanol were added to each well for 15 min to fix the biofilm, and then the supernatants were discarded. Microplates were air-dried, and then 100 µL of crystal violet solution (1:50 from stock solution) were added to wells and incubated at room temperature for 20 min. The extra dye was washed away with tap water, and then 150 µL of acetic acid 33% were added to the wells. The absorbance was measured at 590 nm using a Thermomax Molecular Device microplate reader. In parallel, the viable cells in biofilm were assessed by the colorimetric assay that investigates the metabolic reduction of XTT to a water-soluble brown formazan product. In this sense, 100 µL of the XTT/menadione solution (4 mg XTT in 10 mL prewarmed PBS were dissolved and supplemented by a 100 µL menadione stock solution, which contained 55 mg menadione in 100 mL acetone) were added to all wells and incubated in the dark at 37 °C for 3 h. The contents of the wells were transferred to microcentrifuge tubes and centrifuged at 4000× *g* for 5 min. A total of 100 µL of supernatant from each well was transferred to a new microplate, and the colorimetric changes were measured at 492 nm using a microplate reader [45].

### 2.14. Effects of Lopinavir on In Vitro Interaction with Epithelial Cells

Larynx carcinoma cells (HEp2), purchased from RJCB Collection (UFRJ, Brazil), were grown in DMEM supplemented with 10% FBS at 37 °C in an atmosphere of 5% CO_2_, until they reached confluence and then sub-cultivated at least once a week. Prior to the interaction with the yeasts, the HEp2 cells were plated onto 24-well multidishes in the presence of FBS and incubated at 37 °C for 24 h. After this period, the epithelial cells were washed in Hank’s solution and counted in an inverted phase-contrast microscope (Zeiss). For the interaction assay, untreated and lopinavir-treated fungi were added to the monolayer in a ratio of 10:1 (fungi:epithelial cell), and the systems were incubated at 37 °C in 5% CO_2_ for 1 h. Systems were washed with PBS, fixed in Bouin’s solution and stained with Giemsa. The percentage of infected epithelial cells was determined by randomly counting at least 200 animal cells in each duplicate through microscopic examination using an immersion objective in a Zeiss Axioplan 2 microscopy. The association index was obtained by multiplying the percentage of infected epithelial cells by the number of fungi per each infected animal cell [46].

### 2.15. BALB/c Mice for In Vivo Infections

Isogenic females of BALB/c mice (6–8 weeks old) were bred at the University of São Paulo (USP; São Paulo, Brazil) in an animal facility under specific pathogen-free conditions, constant temperature, light and dark cycles 12/12 h, *ad libitum* feeding and the supervision of a single person trained and specialized in laboratory animal care. Procedures involving animals and their care were conducted according to the local ethics committee and international rules. All experiments were approved by the Institutional Animal Care and Use Committee of Institute of Biomedical Sciences (ICB) of USP (number 042-127-02). When the survival assay was carried out, rigorous monitoring of infected animals was done twice per day in order to detect any signs of advanced disease, pain or distress. No mice presented some of these symptoms. Some animals died during the night with a rapid evolution of the disease; in this case, the mice were removed early the next day in order to avoid the stress of the other animals, following the guidelines of the Guide for the Care and use of Laboratory animals.

### 2.16. Effects of Lopinavir on In Vivo Infection of Immunocompetent Mice

Six female BALB/c mice per group were intravenously infected with 1 × 10^5^ yeasts of *C. albicans*. After 1 h of fungal infection, the animals were treated with PBS (control group), fluconazole (at both 10 and 15 mg/mL) and lopinavir (at both 10 and 15 mg/mL). The mice survival was evaluated every day during 5 consecutive days, and no animals died during this period. To evaluate the fungal burden, after 5 days post-infection, the animals were anaesthetized with 80 mg/kg ketamine and 10 mg/kg of xylazine and then euthanized by cervical dislocation. Subsequently, the kidneys, spleen and liver of the mice were dissected aseptically, weighed and homogenized in 1 mL of sterile PBS. Aliquots of the homogenate (100 μL) were inoculated with a BHI medium containing 2% agar. After incubation for 24 h at 37 °C, the number of CFUs was determined. The effectiveness of treatment was determined by comparing the number of CFUs per gram of tissue of treated animals with the number of CFUs per gram of tissue of control animals (PBS-treated animals) [47].

### 2.17. Effects of Lopinavir on In Vivo Infection of Immunosuppressed Mice

For immunosuppression of the animals, initially, doses of 100 mg/kg cyclophosphamide were administered intraperitoneally 4 days and 1 day before infection with *C. albicans*. In addition, 3 days after fungal infection, another dose of cyclophosphamide was applied in order to ensure the animal immunosuppression [48]. The animals were kept in cages lined with wood shavings and closed with autoclaved filter, and served autoclaved food and water in order to maintain a sterile environment. Cages were exchanged twice a week in laminar flow hoods. The animals were considered anergic when the number of leukocytes was found to be less than 100 cells/mm^3^ [49]. Six immunosuppressed female BALB/c mice per group were intravenously infected with 1 × 10^5^ yeasts of *C. albicans*. After 1 h of fungal infection, the animals were treated with PBS, fluconazole (10 mg/mL), lopinavir (10 mg/mL) and a combination of fluconazole and lopinavir (each drug at 10 mg/mL). The mice survivability was evaluated every day during 5 consecutive days and, under the employed experimental conditions, some animals died, which allowed the construction of a survival curve. Animals that survive after 5 days post-infection were anaesthetized and then euthanized by cervical dislocation. Then, the kidneys, spleen and liver of the mice were dissected aseptically, weighed, homogenized in 1 mL of sterile PBS and spread onto BHI agar plates to measure the CFUs. In parallel, the excised kidneys were also fixed in 10% buffered formalin and dehydrated with increasing concentrations of ethanol. The samples were then treated with xylene and embedded in paraffin. Tissue sections of 3 μm were stained with Grocott-Gomori’s methenamine silver staining and then examined with an Axio Imager M.2 optical microscope (Zeiss, Jena, Germany).

### 2.18. Effects of Lopinavir on the Expression of Sap1-3 Antigens in Post-Mice Passage

In this set of experiments, the expression of Sap1-3 antigens, which are well-known virulence factors that directly influence the course of infectious process, was compared before and after passage of *C. albicans* in the murine model. To do it, ten yeast colonies isolated from the kidneys of infected mice, which were treated with PBS, fuconazole (10 mg/mL), lopinavir (10 mg/mL) and fluconazole + lopinavir (each drug at 10 mg/mL) as explained above, were randomly selected from the BHI plate, washed in PBS and grown in the YCB-BSA medium at 37 °C for 48 h under agitation (100 rpm). Subsequently, the fungal cells were processed to evaluate the expression of the Sap1-3 antigen by flow cytometry as earlier described in the item 2.11. As a control, *C. albicans* (strain 11) yeasts only grown in axenic conditions (and never used in in vivo animal infection) were also evaluated to determine the basal level of Sap1-3 expression.

### 2.19. Statistics

All experiments were performed in triplicate, in three independent experimental sets. The results were analyzed statistically by Student’s *t*-test (in the comparisons between two groups) and by the Analysis of Variance One-Way ANOVA followed by a Tukey-Kramer post-test (in comparisons between three or more groups). In the case of the survival curve, the Log-rank (Mantel–Cox) test was used to indicate statistical significance. In all analyses, *p* values of 0.05 or less were considered statistically significant. All analyzes were performed using the program GraphPad Prism version 6.0 (GraphPad Software, San Diego, CA, USA).

## 3. Results and Discussion

### 3.1. Lopinavir Binds to Sap2

It is well-known that *C. albicans* possesses ten distinct *Sap* genes (*Sap1* to *Sap10*). Saps are classical virulent factors, whose expressions are modulated and/or influenced by the physicochemical environmental conditions, such as pH, temperature, nutrient availability and site of infection [50,51]. Sap2 is the best-studied Sap isoenzyme of *C. albicans*; since it is constitutively expressed, it is the dominant Sap isoenzyme in both in vitro and in vivo experimentations; it has broad substrate specificity, and it participates in distinct phases of *C. albicans*-host interplays [50,51]. Moreover, individuals with candidiasis have high titers of antibodies against Sap2, with soluble antigens present in their serum [52]. Based on those multimodal roles, Sap2 of *C. albicans* is considered a druggable target to new antifungal drugs [53,54]. So, Sap2 was selected to evaluate the ability of lopinavir, a clinically available HIV PI [55], in binding to its catalytic site by an in silico approach.

First of all, a comparison of the docking of the ligand A70450 to Sap2 was taken as a benchmark. The protonation states of Sap2 D_32_ and a potential quaternary nitrogen atom of the ligand were probed, resulting in four docking runs: protonated ligand and D_32_, unprotonated D_32_ and ligand, protonated ligand and unprotonated D_32_ and unprotonated ligand and protonated D_32_. The protonated D_32_ and A70450 run was the only combination capable of reproducing the experimental geometry of the complexed ligand to Sap2 (Figure S2). The protonation of the nitrogen atom in the ring of A70450 is necessary due to the vicinity of the side chain carboxylate group of D_120_ to the ring (Figure S2). This effect is therefore expected to play a minor role in the smaller ligand lopinavir.

**Figure 1:**
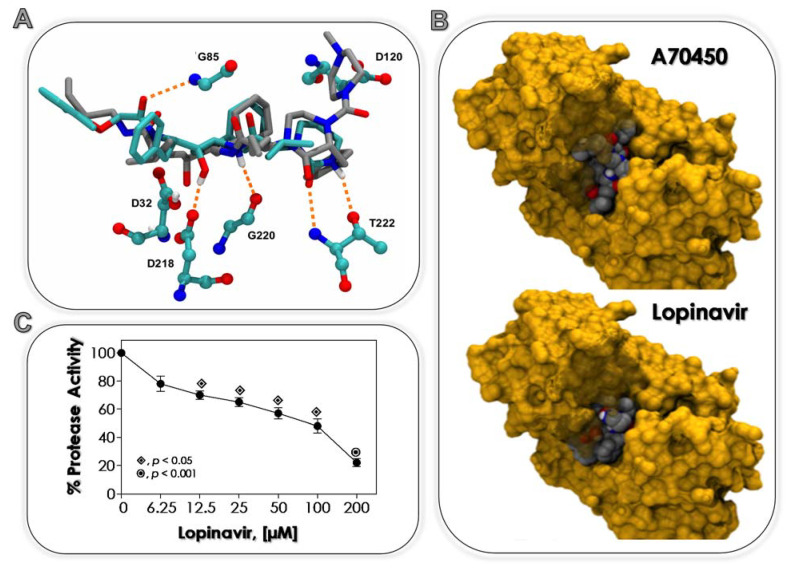
(**A**) Comparison of the geometries of the docked A70450 to *C. albicans* Sap2 catalytic site as determined experimentally (carbon atoms in gray) and the best binding mode for lopinavir (unprotonated form) by molecular docking (carbon atoms in cyan). Potential hydrogen bonds are shown by orange dashed lines. Remaining atoms are color-coded as oxygen: red, nitrogen: blue, hydrogen: white. Except for the polar hydrogen of D_32_, the remaining protein hydrogen atoms were removed for clarity. (**B**) Molecular docking of A70540 and lopinavir. The protein is represented by its molecular surface in yellow and the ligands in CPK model (atoms are color-coded as carbon: gray, oxygen: red, nitrogen: blue; hydrogen: white). (**C**) Effects of lopinavir on the Sap activity detected in conditioned supernatant of *C. albicans*. Fungal supernatant was incubated in the absence and in the presence of different concentrations of lopinavir, and then the proteolytic activity was measured spectrophotometrically using BSA as protein substrate. The values represent the mean ± standard deviation of three independent experiments performed in triplicate. The symbols highlight the statistically significant differences (Student’s t-test) between untreated and lopinavir-treated systems.

Taken together, the estimated pKa of D_32_ and the above results, this residue was considered in its protonated form for the dockings with lopinavir. The docking results are summarized in Table 1, where the relative binding free energies for the geometries where the ligands interact with D_32_, D_218_ and G_85_ are listed along with their ranked position. Cluster rank 1 means the best binding mode, while 9 represents the worst binding mode, within an estimated 3 kcal/mol maximum difference. In fact, the protonation state of the nitrogen atom of the ring in lopinavir did not seem to be energetically decisive in binding Sap2. However, taking into account the most prevalent conformation and the energetic criterion, it suggested that lopinavir binds preferentially in its unprotonated form (Table 1). This finding seems consistent with the absence of negatively charged residues in the vicinity of this ring in lopinavir. Its docked geometry reveals a very similar binding mode to A70450 (Figure 1A,B). All residues interacting with lopinavir do also interact with A70450 (G_85_, D_32_, D_218_, G_220_ and T_222_) (Figure 1A). In addition, both ligands bind to *C. albicans* Sap2 with very similar binding free energies (difference is within the method uncertainty).

Molecular docking methods provide an approach to the ranking of potential ligands with respect to their ability to interact with a given target [56]. It involves efficient sampling of possible poses of the small molecules in the specified binding pocket of a protein in order to identify the optimal binding geometry, as measured by a score function [57]. The results presented herein revealed for the first time the ability of the HIV PI lopinavir to bind to the catalytic site of Sap2 produced by *C. albicans*. A simple explanation for this result is based on the fact that both HIV-1 protease and Sap2 belong to the same superfamily of aspartic proteases. In addition, the tridimensional structure superimposition of Sap2 and HIV-1 protease confirmed the similarity between their active sites and flap regions [58]. However, while many chemical interactions are common to the bind of lopinavir to both viral and fungal aspartic proteases, the different electrostatic and hydrophobic contributions in each active site will lead to distinct inhibitory potential. In this sense, distinct HIV PIs have similar Ki against HIV-1 protease at the nanomolar range, while the affinity for Sap2 of *C. albicans* is much lower usually at the micromolar range [53,59].

A detailed work published by Calugi et al. [58] described that both ritonavir and saquinavir, two first-generation HIV PIs, were able to interact with both catalytic D_218_ and D_32_, as observed in the HIV-1 protease complex, and another hydrogen-bond is established between the main chain carbonyl moiety and G_85_ amide proton. That result is similar to which was observed herein with lopinavir, a second-generation HIV PI. Additionally, docking results showed that saquinavir addressed S2/S2′ and S1/S1′ Sap2 binding pockets, while ritonavir [58] and lopinavir (present work) addressed the S3 subsite of the Sap2 active site. Based on the structure of Sap2 of *C. albicans* complexed with the standard inhibitor A70450, it has been proposed that binding to the S3 region is critical for the efficient inhibition of this enzyme [60]. Interestingly, ritonavir also bound to the Sapp1p active site of *Candida parapsilosis* in an extended conformation and occupied the S2-S3′ enzyme substrate binding subsites, and the hydroxyl group of the central ritonavir moiety formed two hydrogen bonds with the side-chains of the two catalytic aspartates D_32_ and D_220_ [61].

### 3.2. Lopinavir Inhibits Sap Activity

It is well-known that *C. albicans* cells are able to secret Saps to the extracellular environment when grown in a chemically defined medium supplemented with a single protein source, such as albumin and hemoglobin [26]. Corroborating this statement, it was showed that *C. albicans* strain 11 (which was used in all parts of the present study) secreted aspartic-type proteases, particularly Sap2, as judged by the ability to degrade soluble BSA, the inhibition of the proteolysis by pepstatin A (a prototypal inhibitor of Saps) and the recognition by the anti-Sap antibody (Figure S1) [33]. As experienced in the present work, lopinavir was able to inhibit the BSA degradation in a typically concentration-dependent manner (Figure 1C), showing an IC_50_ of 58.7 µM.

As is well documented in the current literature, HIV PIs have been described as potential inhibitors of Sap produced by clinically relevant fungi. For example, amprenavir blocked *C. albicans* Sap activity, whose inhibition increased from 85% to 100% as drug concentration increased from 6.25 to 200 µM [33]. Indinavir at 1 µM reduced the *C. albicans* Sap activity by more than 50%, increasing the inhibitor concentration to 10 µM, completely abolishing the proteolysis [62]. Indinavir also restrained the aspartic protease secreted by *Cryptococcus neoformans* yeasts [63]. Valle et al. [64] showed that seven out of nine HIV PIs significantly reduced the hydrolytic activity of Sap-like enzymes produced by *Trichosporon asahii*, presenting inhibition profiles ranging from 52 to 67% (atazanavir > nelfinavir = ritonavir = lopinavir > indinavir = amprenavir > saquinavir), while tipranavir and darunavir did not inhibit the proteolysis. Mycelial [65], conidial [34] and sclerotic [66] cells of the filamentous fungus *Fonsecaea pedrosoi* were able to produce Saps when grown under chemically defined conditions, which were inhibited at different proportions to indinavir, saquinavir, ritonavir and nelfinavir. *Phialophora verrucosa* mycelial cells also extracellularly released aspartic-type protease, which was inhibited by HIV PIs at 100 µM as follows: amprenavir (~77%), ritonavir (~76%), lopinavir (~75%), indinavir (~51%), atazanavir (~46%), saquinavir (~41%) and nelfinavir (~36%) [67]. Collectively, all these results confirm that aspartic proteases produced by opportunistic human fungal pathogens are genuine targets of the HIV PIs.

### 3.3. Lopinavir Interferes with Growth Behavior, Morphometrical Parameters and Morphology

**Figure 2:**
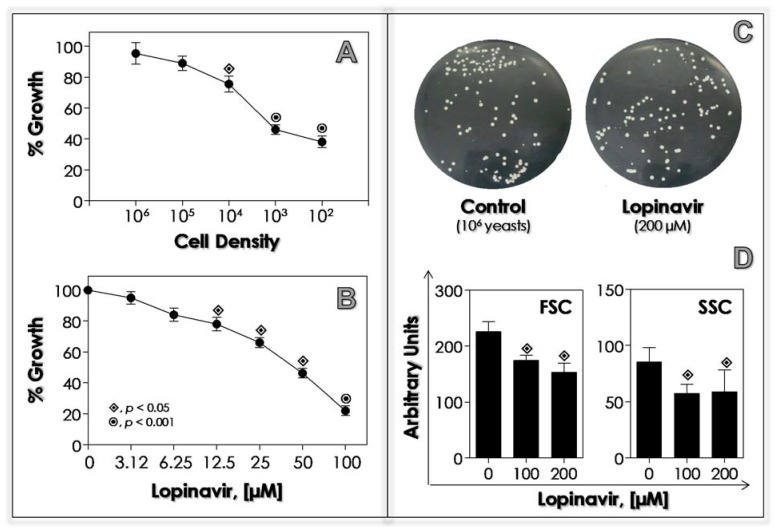
Effects of lopinavir on the growth of *C. albicans* yeast cells. (**A**) Different fungal densities (10^2^–10^6^ yeasts) were treated with lopinavir at 50 µM. (**B**) Yeasts (10^3^ cells) were treated with different concentrations of lopinavir (0–100 µM). In both set of experiments, the yeasts were incubated for 20 h at 37 °C with slight agitation. A control was made replacing lopinavir with PBS, which was considered as 100% of growth. Inhibition ratio was determined by CFU measurements. (**C**) The representative plates demonstrate the treatment of high cellular density (10^6^ yeasts) with lopinavir at 200 µM for 20 h, revealing a similar growth rate in comparison to the untreated ones. (**D**) In parallel, 10^6^ yeasts were treated (or not) with lopinavir (at both 100 and 200 µM) for 20 h and then analyzed by flow cytometry in order to measure size and granularity. Forward scatter (FSC) measurement is related to cell size, and side scatter (SSC) measurement is related to the internal granularity and/or complexity of a cell. In all cases, the values represent the mean ± standard deviation of three independent experiments performed in triplicate. The symbols indicate the experimental systems considered statistically significant from the control (Student’s *t*-test).

Antimicrobial action is directly dependent on two main parameters: inoculum size and drug concentration. Moreover, inoculum concentration (in other words, the number of available targets) may particularly affect antifungal drugs whose antimicrobial activity is based on an enzymatic mechanism [68,69]. In this context, we analyzed the effects of lopinavir using distinct fungal densities and different concentrations of this drug. Lopinavir at 50 µM was able to affect the viability of *C. albicans* considerably in densities ranged from 10^2^ to 10^4^ yeasts; while using both 10^5^ and 10^6^ yeasts, the growth behavior was not statistically different in comparison with untreated fungi (Figure 2A). In addition, 10^6^ yeasts treated for 20 h with a higher lopinavir concentration (200 µM) had no alterations in CFU counts compared to the control cells (Figure 2B). On the other hand, when 10^3^ yeasts were incubated with lopinavir, a typically dose-dependent inhibition on the growth rate was clearly observed (Figure 2C), displaying an IC_50_ of 39.8 µM.

**Figure 3:**
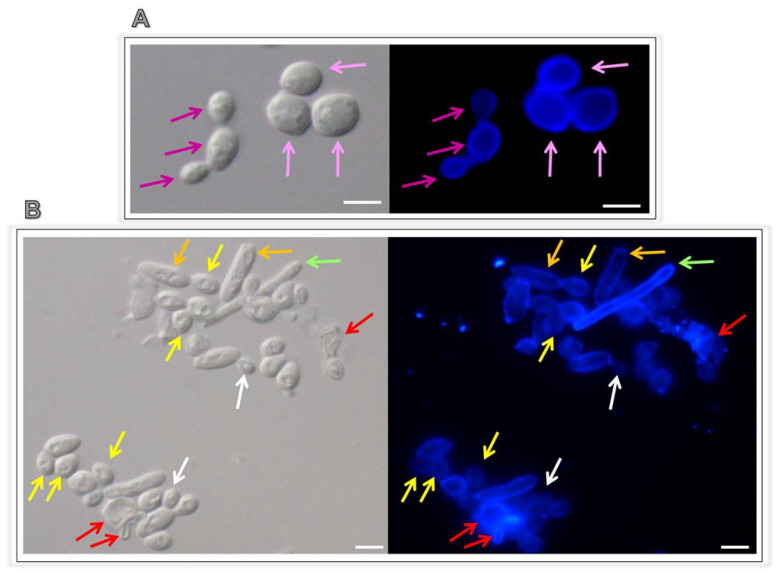
Light and fluorescent microscopy images exemplifying the morphological differences observed in *C. albicans* cells grown in YCB-BSA medium in the absence (**A**) and in the presence of 200 μM lopinavir (**B**) for 20 h. The control cell population is homogeneous and consists of rounded yeast cells (light pink arrows) and pseudohyphae (dark pink arrows). Contrarily, the treatment with lopinavir induces some clear morphological alterations, including petite cells (white arrows), smaller and ovoid cells (yellow arrows), large non-ovoid cells (orange arrows) and ghost or lysed cells (red arrows), as well as some small hyphae (green arrow) were observed. Bars = 5 µm.

Lopinavir induced alterations in fungal morphometrical parameters, causing a significant reduction in cellular size and granularity (Figure 2D). A simple inspection of *C. albicans* cells treated with lopinavir by means of light and fluorescence microscopies revealed some morphological alterations, including non-rounded, petite and lysed cells, compared to the typical appearance of rounded-shape yeasts (Figure 3). Interestingly, lopinavir also induced the appearance of small hyphae in the culture (Figure 3).

Amprenavir was also able to inhibit the growth of *C. albicans* in typically concentration- and number of yeast (target)-dependent ways, but displaying an IC_50_ value three times higher compared to lopinavir [33]. In addition, amprenavir at 100 µM promoted a reduction of 30% in *C. albicans’* size compared with untreated cells [33]. Ritonavir at 8 mg/mL interfered with the consumption of albumin by *C. albicans* and *C. parapsilosis* when cultured in YCB-BSA medium, reducing the growth rate in 50% after 23 and 86 h, respectively, probably by blocking the access to the nitrogen source as previously proposed as the main inhibitory mechanism [26]. This proposal is exactly as expected from the commonly agreed-upon model of the positive feedback mechanism of *Sap* gene expression, which is up-regulated by peptides generated by the initial albumin proteolysis by the constitutively expressed Saps [70,71].

Nonetheless, contrasting results have been published regarding the ability of HIV PIs to act as antifungal drugs. In this context, indinavir did not affect the *C. albicans* viability at concentrations up to 1 mg/mL, while saquinavir (1 mg/mL) was even fungicidal as assessed by three different viability assays (CFU, MTT, propidium iodide staining), and ritonavir at the same concentration significantly affected the mitochondrial activity only [62]. Indinavir, a HIV PI capable of crossing the blood-brain barrier, was vastly tested against *C. neoformans*, since this fungus has a tropism to the brain. Indinavir impaired the *C. neoformans’* growth in typically time- and dose-dependent fashions for both reference strains and clinical isolates as judged by the CFU assay [63]. However, mitochondrial viability (assessed by either MTT or JC-1) and plasma membrane integrity (propidium iodide staining) were not affected after exposure of *C. neoformans* to indinavir at 10 µM [63]. Mata-Essayag et al. [72] reported that all 73 clinical isolates of *C. albicans* were susceptible to saquinavir, while 23 were susceptible to indinavir, 12 to ritonavir and 3 to nelfinavir. Notably, *C. albicans* clinical isolates (*n* = 17) resistant to fluconazole were also susceptible to saquinavir [72].

Ritonavir was able to reduce the in vitro growth of planktonic cells of *Trichosporon asahii* and *Trichosporon inkin* up to 25 μg/mL, causing a reduction of ~100% at 200 μg/mL [73]. Both saquinavir and ritonavir powerfully inhibited the growth of clinical isolates of the dimorphic fungus *Histoplasma capsulatum*, presenting MIC values ranging from 0.125 to 1 µg/mL for saquinavir considering both filamentous and yeast phases as well as MICs from 0.0312 to 4 µg/mL and 0.0625 to 1 µg/mL for ritonavir against filamentous and yeast phases, respectively [74]. Additionally, those authors reported the synergistic interactions among the combinations of itraconazole with saquinavir or ritonavir against *H. capsulatum*, culminating with a significant reduction in the MIC values of these drugs [74]. The growth of *F. pedrosoi* conidial and sclerotic cells was affected by the treatment with HIV PIs (nelfinavir, ritonavir, saquinavir and indinavir), in which indinavir and ritonavir (both at 100 µM) were the best PIs to block conidial viability and saquinavir (100 µM) the only one able to reduce the viability of sclerotic cells [34,41,65]. In parallel, a significant reduction in the cellular granularity was seen in HIV PIs-treated *F. pedrosoi* conidia, but no alteration was observed in the size parameter [41]. Ritonavir was capable of promoting significant growth inhibition of *P. verrucosa*, with a significant decrease of 60, 45 and 40% at 400, 200 and 100 µM, respectively, displaying an IC_50_ of 141.42 µM [67].

### 3.4. Lopinavir Alters Ultrastructural Architecture

**Figure 4:**
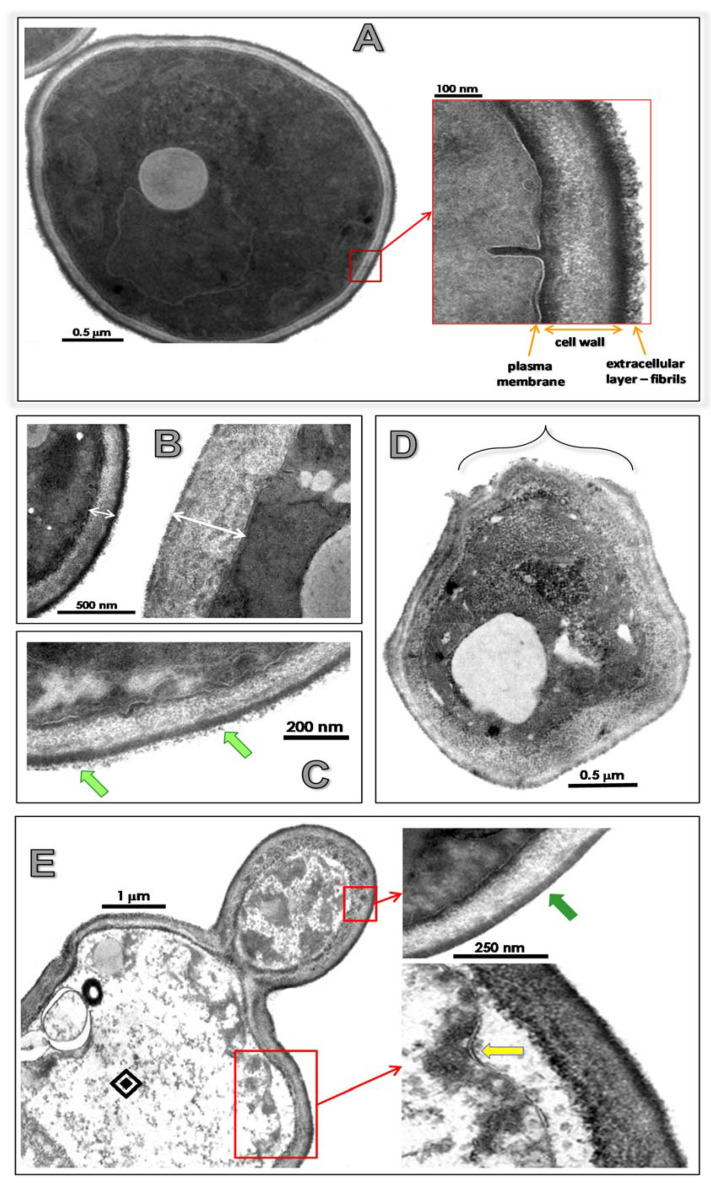
Effects of lopinavir on the ultrastructure of *C. albicans* yeast cells. (**A**) Untreated and (**B**–**E**) lopinavir (100 and 200 µM)-treated fungal cells were processed and analyzed by transmission electron microscopy. (**A**) Control yeasts present a dense cytoplasm with a well-delineated plasma membrane and a compact cell wall, in which the external fibril layer is clearly evidenced (for better visualization, see the amplification of the selected surface area). (**B**) Some lopinavir-treated cells presented a significant augmentation in the cell wall thickness compared to untreated ones (compare the cell wall thickness of the control cell at the left with the lopinavir-treated cell at the right; the cell walls were delimited by white arrows to facilitate the comparison). (**C**,**E**) The external fibril layer is rarefied by the treatment with lopinavir at 100 µM (C—green arrows) and totally lost by lopinavir at 200 µM (E—green arrows). (**D**) The symbol appoints the breakage of cell wall starting from extracellular medium. (**E**) The yellow arrow indicates the invagination of the plasma membrane with consequent withdrawal from the cell wall following treatment of yeasts with lopinavir at 200 µM.

TEM visualization revealed that untreated *C. albicans* yeast cells had a normal morphology with a typical dark and dense cytoplasm, well-delineated plasma membrane and distinct cell wall structure with preserved external fibrils (Figure 4A). Lopinavir promoted significant changes in the surface topography, including (i) augmentation in cell wall thickness (Figure 4B); (ii) reduction (treatment with lopinavir at 100 µM) and total loss (treatment with lopinavir at 200 µM) of the fibrils composing the outermost part of the cell wall (light and dark green arrows, respectively) (Figure 4C,E). Additionally, disorder and detachment of cell wall (Figure 4D), empty cytoplasm resembling ghost cells, plasma membrane presenting numerous undulations and/or invaginations and withdrawal of the plasma membrane from the cell wall were observed in lopinavir-treated fungi (Figure 4E). Taken together, all these significant alterations corroborate the anti-proliferative properties of lopinavir upon *C. albicans*.

**Figure 5:**
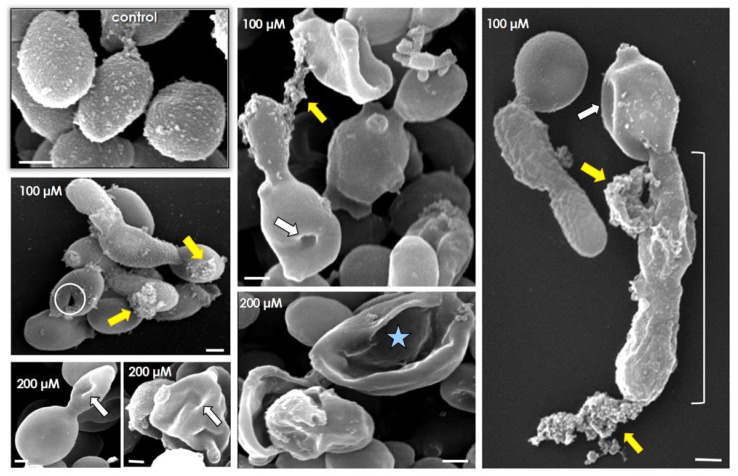
Effects of lopinavir on the ultrastructure of *C. albicans* cells. Untreated and lopinavir (100 and 200 µM)-treated fungal cells were processed and analyzed by scanning electron microscopy. Control cells are characterized by oval-to-round cells with a rough surface, covered by several protrusions. The treatment with lopinavir promoted (i) the detachment of surface materials from the cell wall in both yeasts and filamentous (delimited by brackets) as indicated by yellow arrows; (ii) the conversion of rough surface to smooth one; (iii) surface invaginations in both yeast and daughter cells (white arrows); (iv) alterations on yeast shape, some of them with altered surface sculpturing and (v) lysis of fungal cells (white circle and blue star). Bars = 0.5 µm.

SEM assay was conducted to better reveal the surface modifications provoked by lopinavir treatment in *C. albicans*. As expected, spherical to oval yeasts containing several protrusions (rough surface) were observed in non-treated cells (Figure 5, control). Lopinavir-treated cells presented a smooth surface, some cells presenting a clear detachment of the outside cover (the fibrils) and others containing a focus of attached material, which might represent remnants of the former surface’s irregular external layer (Figure 5, yellow arrows). In addition, lopinavir induced considerable changes in the shape (e.g., yeasts lose their typical oval/round shape) and in the surface sculpturing (e.g., invaginations forming cavitations were usually seen in the surface of both yeasts and daughter cells) (Figure 4, white arrows). Disrupted (lysed) cells, showing only the remaining framework, were also observed, particularly when fungal cells were treated with lopinavir at 200 µM (Figure 5, white circle and blue star). Filamentation was detected in some lopinavir-treated cells; however, this structure presented serious surface injuries such as those described in yeasts (Figure 5, white brackets).

Similar ultrastructural alterations were observed in amprenavir-treated *C. albicans* cells by means of SEM analysis, particularly the conversion of the rough surface in untreated yeasts to the smooth one after the drug treatment [33]. In fact, different HIV PIs promoted analogous ultrastructural alterations in distinct fungi independent of the morphological stage. In this context, treatment with nelfinavir or saquinavir (both at 100 µM) induced several ultrastructural changes in crucial structures/organelles of *F. pedrosoi* conidial cells as visualized by TEM, including: invaginations in the plasma membrane and detachment of the plasma membrane from within the cell wall, disorder and detachment of the cell wall, breakage of cell wall starting from the extracellular environment, shedding of a great amount of electron-dense and amorphous material on the outer side of the cell wall, rupture of internal organelles and presence of large and irregular cytoplasmic vacuoles, some of them containing small vesicles and others so intense that they occupied almost the entire cytoplasm [34,41].

### 3.5. Lopinavir Arrests the Yeasts-into-Hyphae Transformation

**Figure 6:**
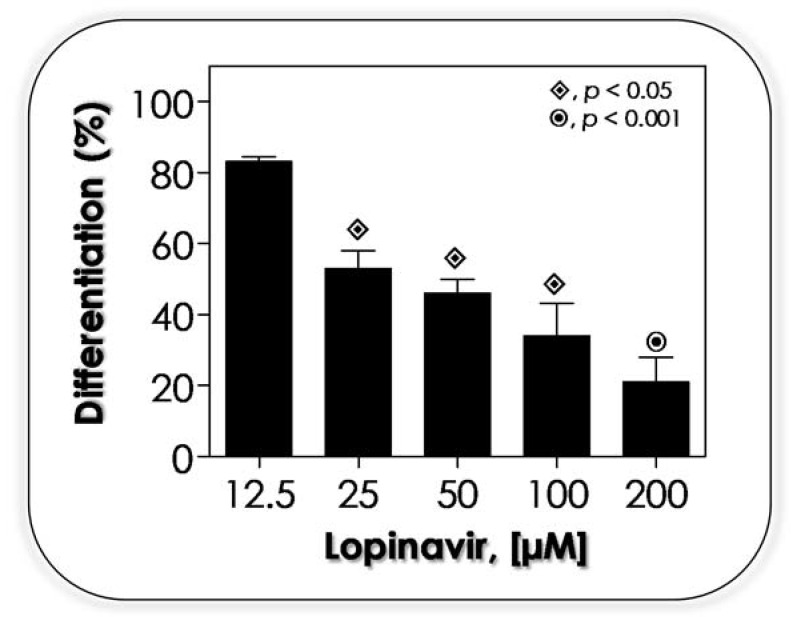
Effect of lopinavir on *C. albicans* yeast into germ tube transformation (morphogenesis process). Germination was performed by incubating the yeasts (10^6^ cells) in fetal bovine serum for 4 h at 37 °C in the absence or in the presence of lopinavir at different concentrations. The number of cells presenting germ tube formation was counted in a Neubauer chamber. The values represent the mean ± standard deviation of three independent experiments performed in triplicate. The symbols indicate the experimental systems considered statistically significant from the control (Student’s *t*-test).

The fascinating ability of fungi to switch between different morphological states is associated with their adaptability, plasticity and pathogenicity. For instance, hyphal growth is able to exert a mechanical force and to secrete a plethora of hydrolytic enzymes, which facilitates the fungal penetration and dissemination inside the host cells and tissues as well as helps to escape from phagocytosis [75,76]. The morphogenesis is a hallmark of the virulence and successfully infectious process of *C. albicans* [77,78]. So, drugs able to arrest the morphogenesis process can be considered promising antifungals. In this context, SEM analysis revealed that lopinavir was capable of causing drastic ultrastructural alterations in both *C. albicans* yeast and filamentous forms (Figure 5). Herein, the effects of lopinavir on yeast into germ tube differentiation (filamentation process) were evaluated using a cellular density (10^6^ yeasts) in which lopinavir was not able to interfere with the fungal viability (Figure 2). The results evidenced that lopinavir arrested the yeast into the germ tube transformation in a concentration-dependent manner (Figure 6).

Gruber et al. [62] described that indinavir did not arrest the ability of *C. albicans* yeasts to form hyphae; however, hyphal elongation of drug-treated cells was delayed by approximately 45% after incubation with indinavir at 0.5 mg/mL. Amprenavir blocked the yeast into the germ tube transformation in *C. albicans* in a typically concentration-dependent way, in which 100 µM of the drug was able to arrest the process by approximately 50% [21]. Saquinavir drastically blocked the *F. pedrosoi* conidia into mycelia transformation observed during the in vitro interaction with epithelial cells [34]. Contrarily, incubation of *C. albicans* with 0.01 µg/mL tipranavir, which is the only non-peptidic HIV PI, favored the in vitro mycelial transition [79].

### 3.6. Lopinavir Disturbs the Synthesis of Neutral Lipids

**Figure 7:**
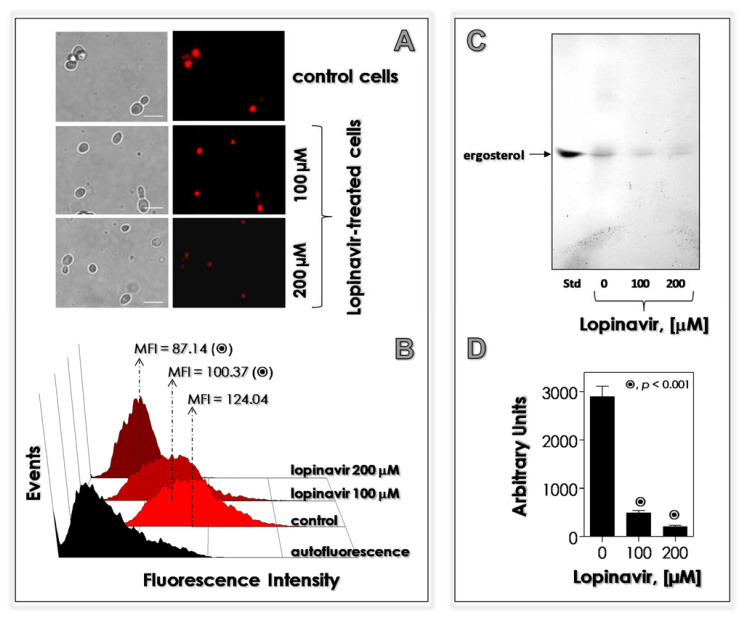
Effect of lopinavir on the production of neutral lipids in *C. albicans*. In this set of experiments, yeast cells were incubated for 20 h in the absence (0 or control cells) and in the presence of lopinavir at both 100 and 200 µM. After that, yeasts were analyzed to detect neutral lipid inclusions (**A**,**B**) and ergosterol (**C**,**D**). (**A**) Differential interference contrast microscopy (left images) and fluorescence microscopy with Nile red (right images) evidenced the intracellular lipid inclusions. (**B**) Flow cytometry assay corroborated the fluorescence microscopy, which showed a drastic reduction in Nile red-positive cells as judged by the drop in the mean of fluorescence intensity (MFI). (**C**) High-performance thin-layer chromatography (HPTLC) showing the ergosterol chemically extracted from the yeast cells as well as the ergosterol standard (Std), which was applied into the same plate. (**D**) Densitometrical analyses of the ergosterol-corresponding bands, expressed in arbitrary units, were shown. The symbols indicate the experimental systems considered statistically significant from the control (Student’s *t*-test).

A common side effect described in individuals who need to use HIV PIs is the interference on the lipid metabolism [80,81]. With this task in mind, we evaluate the effects of lopinavir in the lipid metabolism in *C. albicans*, focusing on the neutral lipids due to their relevance in several crucial processes to yeast cells. These lipids are located in intracellular storages (called lipid bodies) and in plasma membrane (ergosterol) [82]. Our results showed that neutral lipids inside the lipid bodies were drastically reduced by the treatment of yeasts with lopinavir, in a classically dose-dependent way, as judged by both fluorescent microscopy and flow cytometry assays using the fluorophore Nile red (Figure 7A,B, respectively). In consonance, the production of ergosterol was also drastically diminished as revealed by HTPLC assay (Figure 7C). Together, these results can justify the detachment of plasma membrane from the cell wall observed in lopinavir-treated *C. albicans* cells by TEM images, since sterol regulates the plasma membrane fluidity.

Few studies reported the effect of HIV PIs on the lipid metabolism in fungi. Regarding sterol content, untreated conidia of *F. pedrosoi* showed an equal proportion of lanosterol (a precursor of the ergosterol biosynthesis pathway) and ergosterol (the final product) [41]. Contrarily, no ergosterol was detected in nelfinavir-, indinavir-, ritonavir- and saquinavir-treated *F. pedrosoi* conidial cells, whereas lanosterol production was inhibited by saquinavir, ritonavir (~60%) and nelfinavir (~40%), but not by indinavir [41]. The treatment of *C. albicans* cells with amprenavir at 200 µM hugely reduced (90%) the amount of plasma membrane-located ergosterol [33].

### 3.7. Lopinavir Modulates the Surface-Located Molecules

SEM images clearly revealed a detachment of external fibrils from the surface of lopinavir-treated *C. albicans* cells, which is a place rich in glycomolecules and enzymes (Figure 5), that suggests a perturbation in this vital structure directly involved in the interaction (e.g., adhesion events) with the environment [83]. Mannose-containing glycoconjugates (e.g., mannoproteins) were detected in the surface of both untreated and lopinavir-treated yeasts in similar proportions (%FC); however, lopinavir induced a significant reduction in the amount of these glycomolecules as observed by the drop in the MFI parameter (~20 and 70% after treatment with 100 and 200 µM of lopinavir, respectively) in the ConA-labeled cells (Table 2). A similar profile was detected when the yeast cells were incubated with the anti-Sap1-3 antibody, revealing a decrease in the Saps located at the surface of lopinavir-treated cells (Table 2). Sialic acid-containing glycoconjugates were negatively altered considering both %FC and MFI parameters due to the treatment with lopinavir (Table 2). Contrarily, lopinavir promoted a substantial increase in the production of *N*-acetylgucosamine-rich molecules in *C. albicans* (Table 2).

Indinavir showed a dose-dependent decrease in cell-bound Sap2 in *C. albicans* as judged by flow cytometry using an anti-Sap2 monoclonal antibody, for instance, reducing around 30% when yeasts were grown in the presence of indinavir at 0.05 mg/mL [62]. Amprenavir (at both 100 and 200 μM) also reduced the expression of surface molecules in *C. albicans*, including Sap antigens and mannose- and sialic acid-rich glycoconjugates [33]. The pre-exposure of *T. asahii* and *T. inkin* cells to ritonavir at 100 μg/mL for 14 days reduced the cell surface hydrophobicity, which is considered a predictor for adhesion and biofilm formation. Flow cytometry analyses demonstrated that indinavir, saquinavir, ritonavir and nelfinavir significantly diminished the percentage of fluorescently ConA-labeled *F. pedrosoi* conidial cells by approximately 54, 32, 22 and 20%, respectively, when compared to untreated conidia, as well as dropped the MFI values, which indicated a reduction in the surface amount of mannose-rich glycoconjugates. In contrast, the sialic acid expression was not significantly altered in *F. pedrosoi* by the treatment with the HIV PIs. Interestingly, indinavir and ritonavir diminished the production of surface melanin in *F. pedrosoi* conidia, which led to the exposition of internal (which were hidden) molecules such as glucosylceramide [41]. These data highlight the anti-virulence properties of HIV PIs. Efficient antifungal drugs need to be capable of blocking at least some of their virulence markers.

### 3.8. Lopinavir Diminishes the Secretion of Hydrolytic Enzymes

The secretion of molecules by fungal cells is a process dependent on the plasma membrane and cell-wall integrity [84]. Moreover, fungal viability depends on the secretion of hydrolytic enzymes to degrade extracellular macromolecules and to interact with abiotic/biotic structures present in the environment [85]. In addition to secreting aspartic proteases as earlier reported, *C. albicans* strain 11 was also able to secrete esterase, but not phospholipase, under the herein employed experimental conditions. Lopinavir treatment significantly diminished the secretion of both aspartic protease and esterase activities (Table 3).

Indinavir, ritonavir, amprenavir and tipranavir promoted a powerful dose-dependent inhibition of Sap production in *C. albicans* cultured in a defined medium containing BSA as the sole nitrogen source, at an acidic pH [33,79,86]. Indinavir-treated *C. neoformans* significantly affected the production of capsular polysaccharide, aspartic-type protease and urease, but not phospholipase and melanin [63,87]. Furthermore, the treatment of both *C. neoformans* and *C. albicans* yeasts with tipranavir significantly reduced the phospholipase activity [79]. Regarding *C. neoformans*, tipranavir moderately reduced the capsule size, while it had no influence on production of either urease or melanin [79]. Similar results on the reduction of *C. neoformans’* capsule thickness were reported by other HIV PIs, such as saquinavir (170 µg/mL), darunavir (100 µg/mL) and ritonavir (120 µg/mL) [88]. Ritonavir (100 µg/mL) blocked the production of extracellular aspartic proteases in *T. asahii* and *T. inkin* after growth for 48 and 72 h [73]. The pre-treatment of *F. pedrosoi* conidia with HIV PIs, particularly saquinavir, ritonavir and nelfinavir, notably arrested the secretion of pepstatin A-sensitive aspartic protease, inhibiting this activity by around 50, 40 and 30%, respectively [41]. Additionally, ritonavir and nelfinavir inhibited lipolytic activities (esterase and phospholipase) by around 70%, while indinavir and saquinavir restrained these substrates’ hydrolyses by approximately 30% [41].

### 3.9. Lopinavir Influences the Biofilm on Abiotic Surface

**Figure 8:**
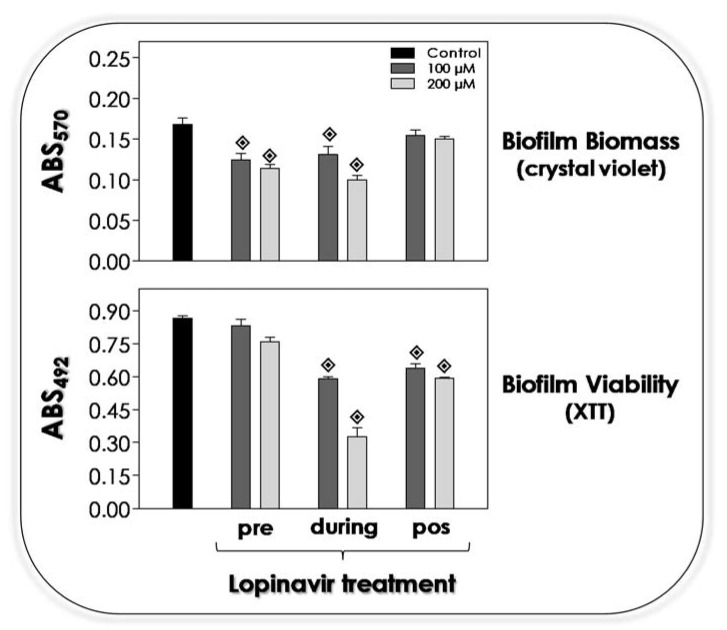
Effects of lopinavir on the biofilm of *C. albicans*. Yeasts were submitted to distinct lopinavir treatment as follows: fungi were pre-treated or not with lopinavir at 100 or 200 μM for 20 h and then added to the polystyrene substrate for additional 48 h (pre); fungi were plated at the same time to adhere to the polystyrene substrate in the absence or in the presence of lopinavir and then the systems were incubated for 48 h (during); and fungi were first adhered to polystyrene for 48 h, and then the mature biofilm was treated with lopinavir for additional 20 h (pos). Subsequently, the spent media were aspirated, and non-adherent cells were removed by thoroughly washing the biofilms with PBS. Biofilm was quantified using two methods to measure biomass (crystal violet staining and quantified by measuring the absorbance at 570 nm) and viability (XTT staining and quantified by measuring the absorbance at 492 nm). Wells with media and no cells were included as blank wells. The values represent the mean ± standard deviation of three independent experiments performed in triplicate. The symbols indicate the experimental systems considered statistically significant from the control (*p* < 0.05; Student’s *t*-test).

It is well recognized that either surface or secreted molecules are directly implicated with the adhesion of *C. albicans* [84,89]. As reported herein, the treatment of *C. albicans* yeasts with lopinavir drastically alters the surface architecture, for example, removing glycomolecules involved with adhesive events to both abiotic and biotic substrates and interfering on the secretion of molecules, including hydrolytic enzymes. Corroborating all these premises, in the present study, the treatment with lopinavir decreased the ability of *C. albicans* to form a robust biofilm over a polystyrene surface, as judged by the significant reduction in the biomass parameter when yeasts were pre-treated or co-incubated with lopinavir at both tested concentrations (Figure 8, upper graphic). However, mature biofilm was not disarticulated by the lopinavir treatment (Figure 8, upper graphic). Regarding the metabolic activity of the fungal cells forming the biofilm, lopinavir was able to reduce the viability when incubated along all of the biofilm formation period as well as when added to the mature biofilm (Figure 8, lower graphic).

The adhesion to abiotic surfaces is the first step to microbial colonization and biofilm formation [90]. Tsang and Hong [91] reported that the pre-treatment of *C. albicans* yeasts with saquinavir (100 µM), ritonavir (100 µM) and indinavir (20 µM) reduced 50% of the adhesion to acrylic strips, which is a common component of oral appliances, that was pre-incubated with pooled human saliva. Amprenavir-treated *C. albicans* cells had their ability to form biofilm biomass reduced in 50% compared to non-treated yeasts [33]. Ritonavir (100 µM) was able to arrest the biofilm formation in both *T. asahii* and *T. inkin*. In that last study, after 48 h of biofilm formation in the presence of ritonavir, fungal viability was drastically diminished (95%) in all *Trichosporon* strains used, while biomass was reduced in 65% for *T. inkin* and 79% for *T. asahii*. However, ritonavir did not alter the viability of mature biofilm formed by *Trichosporon* spp. [73]. Relevantly, SEM images of *Trichosporon* biofilm evidenced a multilayer structure, which was destroyed after the treatment with ritonavir, showing weakly associated sparse cells. Confocal laser scanning microscopy permitted the visualization of the 3D biofilm architecture, which revealed that ritonavir (100 µM) promoted a significant diminution in the biofilm thickness of *T. asahii* (83%) and *T. inkin* (53%), leading to the formation of thinner and fragile biofilms [73].

### 3.10. Lopinavir Blocks the In Vitro Adhesion to Epithelial Cells

**Figure 9:**
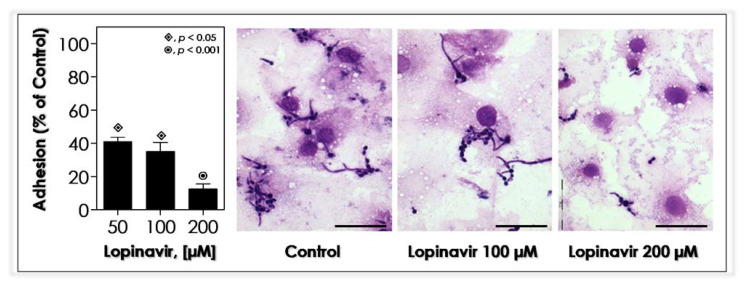
Effect of lopinavir on the interaction process between *C. albicans* and epithelial cells (HEp-2 lineage). In this set of experiments, untreated and lopinavir-treated yeasts were added to the monolayer in a ratio of 10:1 (fungi:epithelial cell), and then the systems were incubated at 37 °C in 5% CO_2_ for 1 h. Systems were washed with PBS, fixed in Bouin’s solution and stained with Giemsa. The percentage of infected epithelial cells was determined by randomly counting at least 200 animal cells in a light microscopy. The values represent the mean ± standard deviation of three independent experiments performed in triplicate. The symbols indicate the experimental systems considered statistically significant from the control (Student’s *t*-test). Representative images of the interaction process are shown. Bars = 20 µm.

The precondition for microbial colonization and successful infection is the adherence event to the host surfaces/structures. Due to the extraction of the external fibril layer of yeast cells by the treatment with lopinavir, several relevant molecules related to adhesion were also taken out (e.g., mannoproteins and Saps), resulting in a serious obstacle to *C. albicans’* colonization, such as inability to form biofilm over the inert polystyrene surface (Figure 8). The interaction with host cells is also coupled up to surface *C. albicans* molecules, such as mannose-riche glycoconjugates (e.g., mannoproteins) [89]. So, as already expected, lopinavir-treated yeasts notably reduced their adhesion to HEp-2 epithelial cells compared to non-treated yeasts (Figure 9).

Ritonavir, saquinavir and indinavir were able to block the adhesion process of *C. albicans* yeasts to human vaginal epithelial cancer cell line HeLa S3 by around 55%, 50% and 30%, respectively, when used at 500 µM [92]. Additionally, the addition of ritonavir and saquinavir at 200 µM reduced the adherence of *C. albicans* to Vero cells by approximately 70% and 50%, respectively, while indinavir was completely ineffective to interfere in the adhesion process [93]. Compared to our results, even taking into account that different epithelial cell lines were used, lopinavir was able to inhibit the fungal adhesion process by approximately 60% at a lower concentration (50 µM) than that used to reach compatible inhibitory indices by both ritonavir and saquinavir. In order to evaluate the specificity of the adherence inhibition caused by ritonavir, a competitive binding experiment was conducted, and the addition of ritonavir to a mixture of purified Sap1-3 proteins prior to adding the *C. albicans* yeasts to interact to Vero cells completely abolished the inhibition of adherence promoted by the treatment with ritonavir alone [93]. Furthermore, saquinavir (0.3 µM) drastically attenuated the injuries caused by *C. albicans* infection in an established in vitro model of oral candidiasis based on reconstituted human epithelium, reducing the cytopathic effects such as vacuolation, edema and detachment of the epithelial layers, as well as fungal invasion [94]. If HIV PIs inhibit fungal adherence, they might prevent candidiasis, since without attachment, the growth rate of *C. albicans* could be insufficient (making the *quorum sensing* signaling unfeasible) to maintain the tissue colonization and to provoke the tissue damage; also, the fungal cells would simply be washed away in the fluids that constantly bathe the mucosal membranes. Corroborating these findings, *C. albicans* mutants that are less adherent are also less virulent [95]. Consequently, a drug able to block the adhesion event could be an attractive anti-virulence and antifungal agent.

The pre-exposition of *C. neoformans* yeasts to indinavir (10 µM) for 72 h followed by the interaction with murine microglial cell line BV2, used as prototype of immune effectors’ cells, culminated in a higher phagocytosis extent compared to the untreated yeasts. In parallel, phagocytosed yeasts were killed by BV2 cells in a higher proportion than the untreated yeasts [63]. In this same line of research, Monari et al. [87] reported that both peripheral blood mononuclear cells and polymorphonuclear leukocytes killed indinavir-treated *C. neoformans* more efficiently than they killed untreated yeasts, which was a process associated with improved production of reactive oxygen species from both cell types. Interestingly, the treatment of *C. albicans* and *C. neoformans* with tipranavir induced contradictory results regarding the interaction with human neutrophils: *C. neoformans* yeasts were more susceptible to the killing by neutrophils than the untreated yeasts; meanwhile, it did not happen for *C. albicans* yeasts [79].

The pre-treatment of *F. pedrosoi* conidia with nelfinavir, saquinavir, indinavir or ritonavir provoked a typically concentration-dependent inhibition pattern on the interaction with Chinese human ovary (CHO) epithelial cells. Relevantly, saquinavir was the most potent inhibitor of both adhesion and endocytosis processes, where the inhibition increased from 60 to 85% (adhesion index) and 70 to 97% (endocytic index) as saquinavir concentration rose from 50 to 200 µM [34]. Moreover, both adhesion and endocytic indexes were considerable reduced taking into consideration the interaction process of HIV PIs-treated *F. pedrosoi* conidial cells with either fibroblasts or macrophages. HIV PIs also improved the macrophage killing capacity against *F. pedrosoi* [34]. Lopinavir at 100 µM diminished the interaction process between *P. verrucosa* conidia and macrophages at about 50%. In addition, the combination of lopinavir (50 µM) plus ritonavir (12.5 µM) affected the adhesion index, reducing it by approximately 40% [67]. Furthermore, the killing capability of macrophages against *P. verrucosa* conidia was significantly enhanced in the presence of lopinavir and ritonavir, individually and in combination [67].

### 3.11. Lopinavir Contains the In Vivo Infection in Immunocompetent Mice

**Figure 10:**
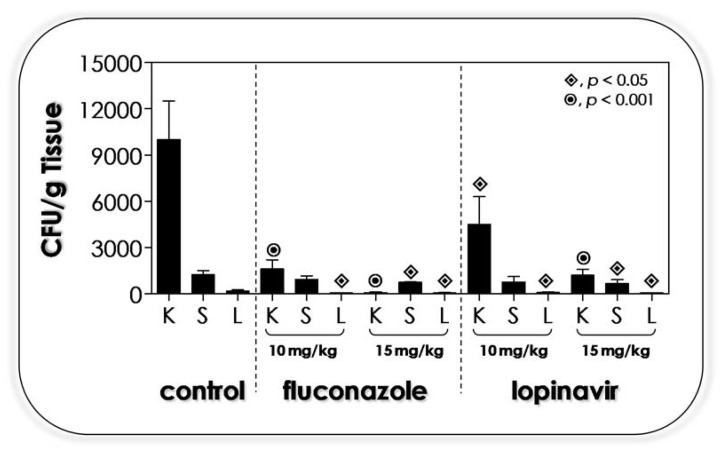
Effects of lopinavir on the in vivo infection of immunocompetent mice with *C. albicans*. Six female BALB/c mice per group were intravenously infected with 10^5^ yeasts. After 1 h, the animals were treated with PBS (control group), fluconazole (at both 10 and 15 mg/mL) and lopinavir (at both 10 and 15 mg/mL). To evaluate the fungal burden, after 5 days post-infection, the animals were anaesthetized and euthanized, and then the kidneys (K), spleen (S) and liver (L) of the mice were dissected, weighed, homogenized in PBS and inoculated in BHI medium to evaluate the number of CFUs. The values represent the mean ± standard deviation of three independent experiments performed in triplicate. The symbols indicate the experimental systems considered statistically significant from the control (ANOVA followed by Tukey-Kramer post-test).

Based on all the in vitro beneficial effects caused by lopinavir on *C. albicans* cells, we decided to evaluate its efficacy on in vivo infection by using a well-established murine model [96]. Firstly, immunocompetent BALB/c mice were intravenously infected with *C. albicans* yeasts, and 1 h after the inoculation, the animals were treated with PBS (positive control of infection), fluconazole (a classical antifungal used to treat candidiasis, including in this animal model) and lopinavir (the test drug). No animals died after 5 days post-infection; therefore, the infection was evaluated by counting the number of CFUs in three target organs: kidneys, spleen and liver. The results demonstrated that fluconazole, even at the lower concentration, was able to decrease the CFU drastically in the kidney, the main target organ in this animal model, when compared to the treatment with PBS (Figure 10). Similarly, lopinavir significantly diminished the infection in the kidney in a dose-dependent fashion. The infection in the liver was controlled by both fluconazole and lopinavir at both employed concentrations (10 or 15 mg/mL). Spleen infection was significantly reduced only when using fluconazole or lopinavir at the highest concentration (Figure 10).

Our study is the first one to demonstrate the efficaciousness of an HIV PI in curing the disseminated infection caused by *C. albicans* in an in vivo animal model. Previously, Cassone et al. [86] showed that indinavir and ritonavir (both used at 10 µM) presented a therapeutic effect in an experimental model of vaginal candidiasis (estrogen-dependent rat vaginitis), with an efficacy comparable to fluconazole, inducing a rapid clearance of the *C. albicans* from the vagina of experimentally infected rats, as shown by the reduction in CFU counts on day 2 after vaginal challenge. Those authors proposed that Sap inhibitors may decisively affect the burden of *Candida* cells present on the vaginal mucosa, and this in turn may favor immunoreconstitution. Corroborating this statement, low burdens of virulent *C. albicans* cells induced a protective response rich in Th1 cells [97]. BALB/c mice intravenously infected with *C. neoformans* and subsequently treated with 14.5 mg/kg of tipranavir or 25 µM of indinavir (intravenously administrated daily for 10 days) showed a significant decrease in CFUs from the brain and liver of animals as observed 15 days after challenge. Interestingly, histological examination of the brain from immunocompetent mice evidenced a smaller capsule size, which is a main virulence factor of this fungal pathogen, in tipranavir-treated mice with respect to the untreated ones [79].

### 3.12. Lopinavir Contains the In Vivo Infection in Immunosuppressed Mice

**Figure 11:**
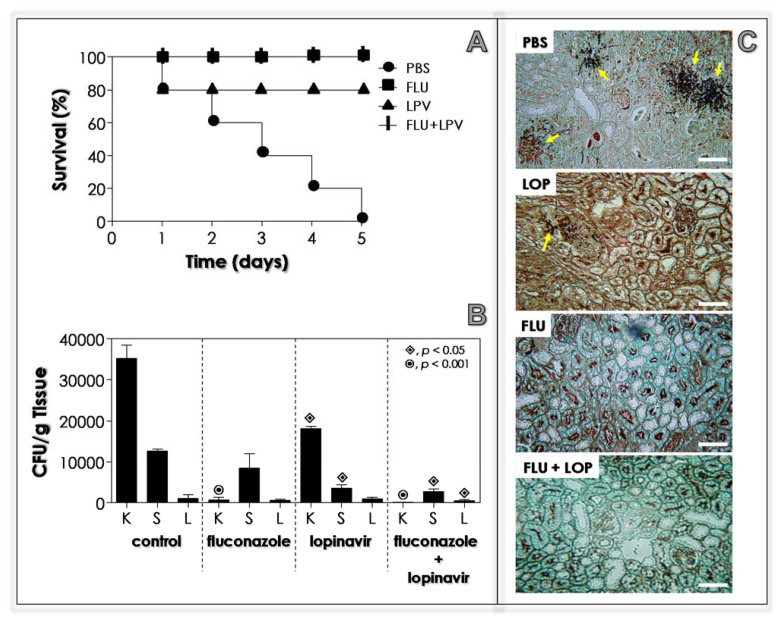
Effects of lopinavir on the in vivo infection of immunosuppressed mice with *C. albicans*. Six cyclophosphamide-immunosuppressed female BALB/c mice per group were intravenously infected with 10^5^ yeasts. After 1 h, the animals were treated with PBS (control group), fluconazole (FLU; 10 mg/mL), lopinavir (LPV; 10 mg/mL) and a combination of both drugs at 10 mg/mL (FLU + LPV). (**A**) The mice survivability was evaluated every day during 5 consecutive days to construct a survival curve. (**B**) To evaluate the fungal burden, after 5 days post-infection, the animals were anaesthetized and euthanized, and then the kidneys (K), spleen (S) and liver (L) of the mice were dissected, weighed, homogenized in PBS and inoculated in BHI medium to evaluate the number of CFUs. Note: The time of animal death occurred throughout the day (some died in the morning, others in the afternoon or in the evening). In this context, the animals were checked every day at three different times: in the morning (at 8:00 a.m.), in the afternoon (at 13:00 p.m.) and in the evening (at 18:00 p.m.) in order to construct the survival curve. So, on the fifth post-infection day, the live animals after the first inspection (in the morning) were selected to analyze the infection on the target organs. The values represent the mean ± standard deviation of three independent experiments performed in triplicate. The symbols indicate the experimental systems considered statistically significant from the control (ANOVA followed by Tukey-Kramer post-test); (**C**) Representative histological images of kidneys after Grocott-Gomori’s methenamine silver staining. The yellow arrows indicate the fungal nests. Bars = 10 µm.

The most problematic issue concerning the candidiasis is related to the host immunosuppressant status. In alignment with this knowledge, we repeated the in vivo infection using cyclophosphamide-immunosuppressed BALB/c mice. Using the same protocol described for immunocompetent animals, we reported a completely distinct pattern of infection, since all the immunosuppressed animals died along the 5 days after being inoculated with *C. albicans* yeasts (Figure 11A). Fluconazole (10 mg/mL) was able to protect all the animals, and no death was detected after 5 days of infection (100% of survival), while lopinavir (10 mg/mL) promoted a rate of 80% survivability (Figure 11A). In animals treated with a combination of fluconazole plus lopinavir, as expected and in consideration of the results herein exposed, this treatment promoted 100% of animals’ survival (Figure 11A). The infection was also monitored by counting the CFUs in the key organs (Figure 11B). Interesting to highlight, the immunosuppression provoked a more robust infection in BALB/c mice (3.5-fold high) in relation to the immunocompetent ones (compare the Figure 10 and Figure 11B, control systems). The drug treatment revealed that (i) fluconazole alone (10 mg/mL) severely reduced the infection in the kidney, but did not change the liver and spleen infections; (ii) lopinavir alone (10 mg/mL) significantly diminished the infections in both kidney and spleen, but not in the liver; and (iii) the fluconazole combined with lopinavir (10 mg/mL of each one drug) resulted in a robust reduction in infection in all evaluated organs (Figure 11B). Histopathology sections of the kidney (the key organ during the *C. albicans* infection in the murine model) corroborated the CFU counts, in which PBS-treated animals presented several nests of fungi compared to fluconazole-, lopinavir- and fluconazole+lopinavir-treated animals (Figure 11C).

Similarly, female immunocompromised BALB/c mice challenged with intravenous inoculation of *C. neoformanas* (10^8^ yeasts) previously exposed to indinavir showed prolonged survival and reduced fungal burden in target organs (liver and brain) with respect to mice infected with untreated *C. neoformans* [98]. Those beneficial results were accompanied by pronounced activation of murine splenic dendritic cells, which induced positive modulation of activatory and costimulatory molecules (e.g., CD86 and CD40) and generated an enhanced secretion of interleukin (IL)-12 and nitric oxide (NO), as well as promoted an increased secretion of IL-2, interferon-γ and proliferation in response to fungal antigens by splenic T cells [98]. Collectively, the reported immunological profile might be a determinant to T lymphocyte cells towards the Th1 response that is considered protective against *C. neoformans* infection [99].

### 3.13. Lopinavir Reduces the Sap Production by Yeasts Recovered from Kidney of Infected Mice

**Figure 12:**
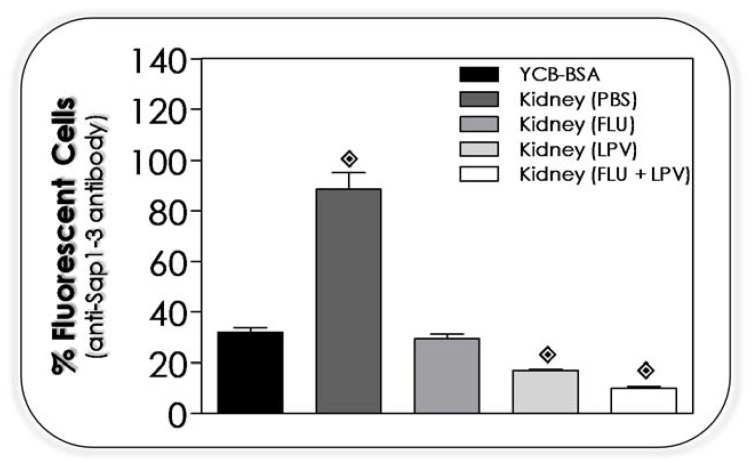
Effects of lopinavir on the expression of Sap1-3 antigens in *C. albicans* before and after passage in BALB/c mice. Yeast colonies isolated from the kidneys of infected mice, which were treated with PBS, fuconazole (10 mg/mL), lopinavir (10 mg/mL) and fluconazole + lopinavir (both at 10 mg/mL) were grown in YCB-BSA medium at 37 °C for 48 h, and then the expression of Sap antigens was evaluated by flow cytometry using an antibody raised against Sap1-3. Yeasts only grown in axenic conditions were run in parallel as a control. The percentage of Sap1-3-labeled yeasts is shown. The values represent the mean ± standard deviation of three independent experiments performed in triplicate. The symbols indicate the experimental systems considered statistically significant from the control (yeasts grown in vitro in YCB-BSA medium) (*p* < 0.05; ANOVA followed by Tukey-Kramer post-test).

Yeasts recovered from kidneys of infected immunosuppressed animals and treated with PBS, fluconazole (10 mg/mL), lopinavir (10 mg/mL) and fluconazole + lopinavir (10 mg/mL of each one drug) were grown in YCB-BSA medium to detect the ability to produce Sap1-3 in comparison to the yeasts not challenged to in vivo infection (Figure 12). Sap1-3 antigen was detected in higher amounts in yeasts recovered from kidneys of animals treated with PBS than the yeasts only cultivated in the in vitro culture medium. Yeasts recovered from animals treated with fluconazole presented the same amount of Sap1-3 antigens compared with yeasts only cultured in in vitro conditions. Relevantly, *C. albicans* yeasts recovered from animals treated with lopinavir or fluconazole + lopinavir had a significant reduction in the production of Sap1-3 antigens compared to the in vitro cultivating yeasts (Figure 12).

An important clinical observation is the decreased occurrence of oral candidiasis in HIV PI-treated individuals [9]. It was speculated that a direct elimination of *Candida* Saps may have a supportive role in this phenomenon, since it has been shown that inhibition of Sap activity by treatment with aspartic PIs resulted in reduced adherence and virulence [59]. Our experiments corroborated this clinical appointment, since *C. albicans* recovered from infected animals treated with lopinavir had a significant decrease in the expression of Sap antigens. In a very interesting work, Cassone et al. [100] compared the level of *Candida* Saps in the saliva of patients receiving two distinct therapies: one group was treated with HAART containing HIV PIs (PI-HAART), and another one treated with HAART containing non-nucleoside reverse-transcriptase inhibitors (NNRTIs). The percentage of Sap-positive, *Candida*-positive subjects and the mean of Sap amount decreased substantially, from 100% to 55% and from 271 to 152 ng of Sap/mL of saliva, respectively, after only 14 days of PI-HAART administration. Along that trend, the Saps were present in only 3 of 8 *Candida*-positive subjects after 30 days, to become undetectable in any subject after 90 and 180 days from treatment initiation. Contrarily, in the NNRTI-HAART group, neither the number of Sap-positive subjects among *Candida*-positive subjects nor the amount of the enzyme was seen to decrease significantly over the duration of treatment [100]. De Bernardis et al. [101] corroborated that HAART-PI, but not HAART-NNRTI, strongly inhibited Sap expression in the oral cavity without exerting any consistent effect on the role of *Candida* spp. isolation or selection of low virulence or antifungal resistant fungus biotype. Early anti-Sap effects and, somewhat later, immunoreconstitution, could favorably interact to make PI-HAART beneficial against candidiasis [102].

A recent work revealed that lopinavir exhibited potent synergistic interactions with azole drugs, particularly with itraconazole, against the emergent, opportunistic and multidrug-resistant *Candida auris* [103]. Those authors studied the mechanisms of action of these drugs working together, and they proposed by using comparative transcriptomic profiling that lopinavir operates as an azole chemosensitizing agent, since it is able to interfere with the glucose permeation and ATP synthesis, which compromised the efficacy of efflux pumps to eject azoles to the extracellular medium; as a consequence, it reverts the well-known azole resistance profile in *C. auris*. Furthermore and similar to our in vivo experiments, the lopinavir/itraconazole combination enhanced the survival rate of *C. auris*-infected *Caenorhabditis elegans* by 90% and reduced the fungal burden in infected nematodes by 88.5% compared to the untreated control [103]. Together, these results endorse that lopinavir is a multimodal drug, operating and triggering multiple responses/actions in biological systems.

## 4. Conclusions

Drug repurposing represents a promising approach to drug discovery, since the identification of novel applications among already existing drugs significantly reduces the time, efforts and expenses needed in comparison to the development of novel drugs. This scenario is particularly important for antimicrobial therapies, once the emergence of multidrug resistant microorganisms is increasingly common, threatening people all over the world and being a challenge to clinicians. As success examples, we can cite the antifungal agent amphotericin B that was latter licensed for leishmaniasis’ treatment [104], and azidothymidine (AZT) that was developed but not approved to treat cancer and was posteriorly licensed for HIV therapy [105,106]. Besides, many other studies have been conducted over time. For example, the antiviral drug valganciclovir has improved the survival of patients with secondary glioblastoma [107], while anti-cancer drugs are promising against bacterial infections [108].

In the present study, by using multiple experimental approaches, it was demonstrated that the action of lopinavir on *C. albicans* attenuated crucial biological events (e.g., nutrition, proliferation, morphogenesis and morphological homeostasis) as well as interfered in processes indirectly/directly related to the establishment of a successful infection (e.g., inhibition of hydrolytic activity of Saps, which are the main virulence factors of this fungus, blockage of synthesis/production of several other virulence attributes, including lipases and mannoproteins, interference on fundamental adhesive surface properties that culminated in inability to form biofilm in inert surface and to interact with epithelial cells). All these in vitro beneficial effects of lopinavir culminated with its ability to control disseminated infection in an in vivo animal model taking into consideration either immunocompetent or immunocompromised mice. Considering the concomitant recurrence of HIV- and *C. albicans*-related infectious diseases and that their major virulence factors, namely HIV-1 protease and *C. albicans* Sap2, respectively, belong to the same superfamily of aspartic proteases, the development of new dual inhibitors able to interact with both targets represents an enormous challenge and a matter of high relevance.

Collectively, our results highlight that lopinavir may be used as promising repurposing drug against *C. albicans* infection as well as may be used as a lead compound for the development of novel antifungal drugs. In this context, the generation of more-specific aspartic PIs with high selective toxicity against Sap-producing fungi would probably represent a valid therapeutic strategy in the battle to get novel (presenting new mechanisms of action) and effective antifungal drugs. Particularly, the beneficial effects of combining lopinavir and azoles (e.g., fluconazole) highlight a promising and relevant line of future research, due to the possibility to reduce the probability of resistant strains emerging and to minimize the drugs’ toxicity and their side effects.

## Figures and Tables

**Table 1:** Molecular docking binding free energies and associated ranking to the different ligands when interacting with *C. albicans* Sap2 catalytic site (D_32_, D_218_ and G_85_).

Ligands	Estimated Binding Free Energy (kcal/mol)	Cluster Ranking
A70450 (standard inhibitor, protonated form)	−9.5	1
Lopinavir (unprotonated)	−9.0	1
Lopinavir (protonated)	−8.6	3

**Table 2:** Effects of lopinavir on the expression of cell surface molecules in *C. albicans*.

[Lopinavir]	Sialic Acid-Rich Molecules (*LFA-Labeled Cells*) ^a^		Mannose-Rich Molecules (*ConA-Labeled Cells*) ^a^		*N*-Acetylglucosamine-Rich Molecules (*WGA-Labeled Cells*) ^a^		Surface-Located Aspartic Proteases (*Sap1-3-Labeled Cells*) ^a^	
	%FC ^b^	MFI ^b^	%FC	MFI	%FC	MFI	%FC	MFI
None	41.96 ± 3.2	542.68	99.22 ± 0.4	1858.30	48.35 ± 1.3	114.62	88.77 ± 0.3	753.58
100 μM	30.75 ± 1.3 *	300.57 **	94.86 ± 3.3	1502.74 *	59.72 ± 0.9 *	161.03 **	90.01 ± 1.7	620.88 *
200 μM	25.30 ± 0.5 **	236.97 **	92.62 ± 2.9	598.31 **	74.04 ± 2.0 **	198.52 **	89.39 ± 1.1	366.67 **

^a^ %LFA, *Limax flavus* agglutinin; ConA, Concanavalin A lectin; WGA, wheat germ agglutinin; Sap1-3; antibody against Sap1-3 of *C. albicans*. ^b^ %FC, percentage of fluorescent-labeled cells; MFI, mean of fluorescence intensity. *, *p* < 0.05; **, *p* < 0.001.

**Table 3:** Effects of lopinavir on the production of extracellular hydrolytic enzymes by *C. albicans*.

Lopinavir	Esterase Activity (*Pz* Value) ^a^	Aspartic Protease Activity (*Pz* Value)
None	0.576 ± 0.011	0.444 ± 0.052
100 μM	0.629 ± 0.038 *	0.595 ± 0.012 **
200 μM	0.672 ± 0.021 **	0.775 ± 0.037 **

^a^*Pz* value means the ratio of the colony diameter per the diameter of colony plus precipitation zone. *, *p* < 0.01; **, *p* < 0.001.

## Data Availability

Not applicable.

## Supplementary Materials

The following are available online at https://www.mdpi.com/article/10.3390/jof7060424/s1, Figure S1. Production of aspartic proteases secreted by *Candida albicans* (strain 11). (A) The enzymatic activity was evidenced after 96 h of in vitro growth in YCB-BSA agar plates by the presence of a large clear zone around the fungal colony, which corresponds to the hydrolysis of the albumin present in the solid medium. (B) The BSA consumption profile along the in vitro growth of *C. albicans* yeasts in YCB-BSA liquid medium was shown. After 24, 48, 72 and 96 h, the fungal cultures were harvested, filtered and the spent culture media were analyzed by SDS-PAGE to demonstrate the cleavage of soluble BSA along the time. A control in which the culture medium was collected before the yeast inoculation was added in the same gel (lane 0). The gel was silver stained and the intact BSA molecule (66 kDa) was shown. Note the gradual BSA fragmentation into smaller peptides. (C) Pepstatin A, a classical aspartic protease inhibitor, at 10 µM fully blocked the BSA cleavage by inhibiting the aspartic protease activities observed in the fungal secretion (line BSA+S+P; compare with the line BSA+S, which represents the incubation of fungal secretions with intact BSA molecules). Line designated as BSA represents the intact BSA molecule incubated only with buffer (pH 4.0). (D) Western blotting assay revealed the presence of a polypeptide of 43 kDa recognized by the anti-Sap1-3 antibody, which corresponds to the molecular mass of Sap2, the main Sap detected in *C. albicans* secretions under the employed conditions (White and Agabian 1995); Figure S2. (A) Standard ligand A70450 and lopinavir in different protonation states used for the molecular docking with Sap2. L1 (umprotonated) and L2 (protonated) correspond to different protonation states of lopinavir. Black arrows highlight the protonated quaternary nitrogen atom lopinavir. (B) Comparison of the geometries of the docked A70450 to SAP2 as determined experimentally (carbon atoms in gray) and by molecular docking (carbon atoms in cyan), when both D32 and the nitrogen atom in the ligand six-membered ring are considered protonated. Sap2 interacting residues are highlighted; potential hydrogen bonds are shown by orange dashed lines. Remaining atoms are color-coded as oxygen: red, nitrogen: blue, hydrogen: white. (Except for the polar hydrogen of D32, the remaining protein hydrogen atoms were removed for clarity).
