# Supplementary material for: Repositioning Lopinavir, an HIV Protease Inhibitor, as a Promising Antifungal Drug: Lessons Learned from Candida albicans—In Silico, In Vitro and In Vivo Approaches
Source: J Fungi (Basel). 2021 May 28;7(6):424. doi: 10.3390/jof7060424 (PMC8229492; doi:10.3390/jof7060424)
Supplement: Supplementary file 1 [file jof-07-00424-s001.zip › jof-1225661-supplementary.pdf]

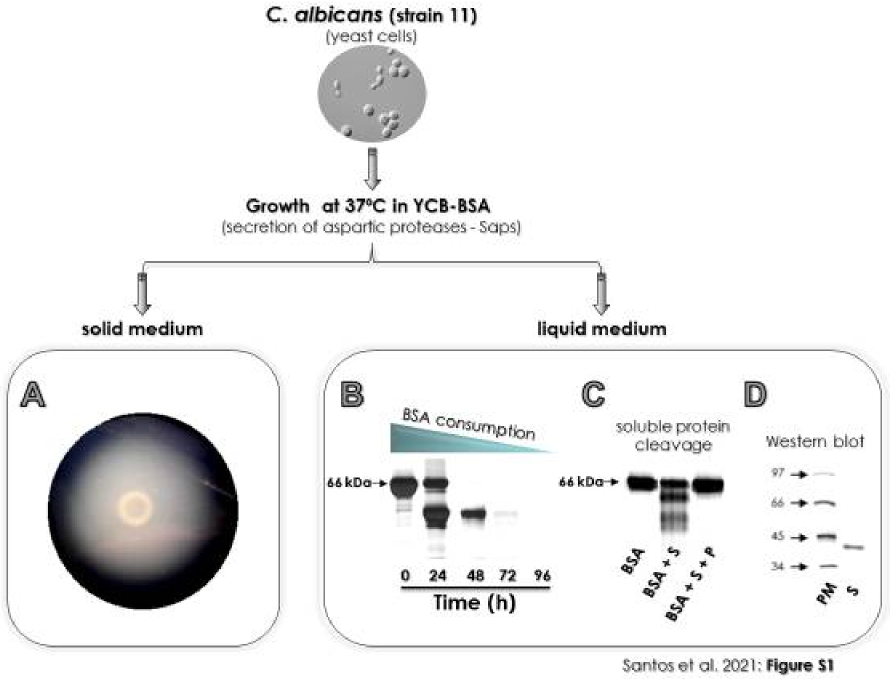

Figure S1. Production of aspartic proteases secreted by *Candida albicans* (strain 11). (A) The enzymatic activity was evidenced after 96 h of *in vitro* growth in YCB-BSA agar plates by the presence of a large clear zone around the fungal colony, which corresponds to the hydrolysis of the albumin present in the solid medium. (B) The BSA consumption profile along the *in vitro* growth of *C. albicans* yeasts in YCB-BSA liquid medium was shown. After 24, 48, 72 and 96 h, the fungal cultures were harvested, filtered and the spent culture media were analyzed by SDS-PAGE to demonstrate the cleavage of soluble BSA along the time. A control in which the culture medium was collected before the yeast inoculation was added in the same gel (lane 0). The gel was silver stained and the intact BSA molecule (66 kDa) was shown. Note the gradual BSA fragmentation into smaller peptides. (C) Pepstatin A, a classical aspartic peptidase inhibitor, at 10 µM fully blocked the BSA cleavage by inhibiting the aspartic peptidase activities observed in the fungal secretion (line BSA+S+P; compare with the line BSA+S, which represents the incubation of fungal secretions with intact BSA molecules). Line designated as BSA represents the intact BSA molecule incubated only with buffer (pH 4.0). (D) Western blotting assay revealed the presence of a polypeptide of 43 kDa recognized by the anti-Sap1-3 antibody, which corresponds to the molecular mass of Sap2, the main Sap detected in *C. albicans* secretions under the employed conditions (White and Agabian 1995).

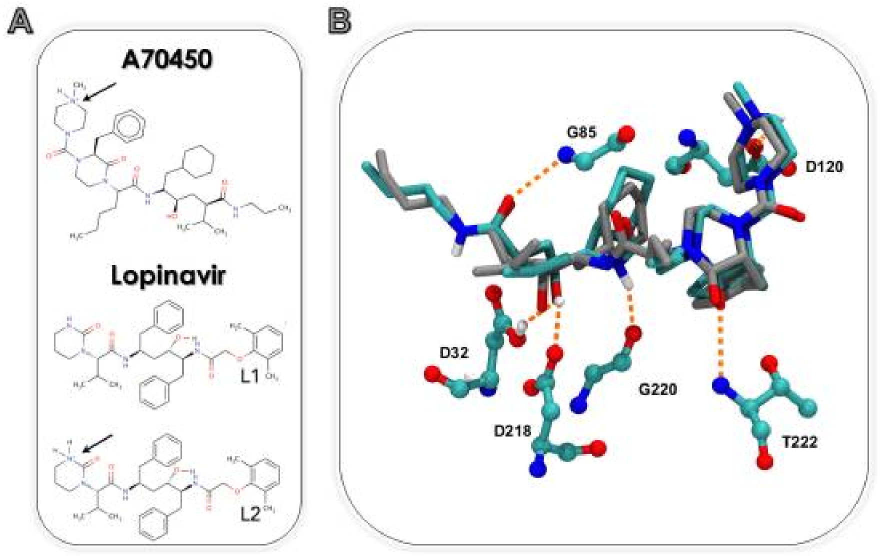

Santos et al. 2021: **Figure S2**

Figure S2. (A) Standard ligand A70450 and lopinavir in different protonation states used for the molecular docking with Sap2. L1 (umprotonated) and L2 (protonated) correspond to different protonation states of lopinavir. Black arrows highlight the protonated quaternary nitrogen atom lopinavir. (B) Comparison of the geometries of the docked A70450 to SAP2 as determined experimentally (carbon atoms in gray) and by molecular docking (carbon atoms in cyan), when both D_{32} and the nitrogen atom in the ligand six-membered ring are considered protonated. Sap2 interacting residues are highlighted; potential hydrogen bonds are shown by orange dashed lines. Remaining atoms are color-coded as oxygen: red, nitrogen: blue, hydrogen: white. (Except for the polar hydrogen of D_{32}, the remaining protein hydrogen atoms were removed for clarity).
